# Preparation by alkaline treatment and detailed characterisation of empty hepatitis B virus core particles for vaccine and gene therapy applications

**DOI:** 10.1038/srep11639

**Published:** 2015-06-26

**Authors:** Arnis Strods, Velta Ose, Janis Bogans, Indulis Cielens, Gints Kalnins, Ilze Radovica, Andris Kazaks, Paul Pumpens, Regina Renhofa

**Affiliations:** 1 Latvian Biomedical Research and Study Centre, Ratsupites Str. 1 k-1, LV-1067, Riga, Latvia

## Abstract

Hepatitis B virus (HBV) core (HBc) virus-like particles (VLPs) are one of the most powerful protein engineering tools utilised to expose immunological epitopes and/or cell-targeting signals and for the packaging of genetic material and immune stimulatory sequences. Although HBc VLPs and their numerous derivatives are produced in highly efficient bacterial and yeast expression systems, the existing purification and packaging protocols are not sufficiently optimised and standardised. Here, a simple alkaline treatment method was employed for the complete removal of internal RNA from bacteria- and yeast-produced HBc VLPs and for the conversion of these VLPs into empty particles, without any damage to the VLP structure. The empty HBc VLPs were able to effectively package the added DNA and RNA sequences. Furthermore, the alkaline hydrolysis technology appeared efficient for the purification and packaging of four different HBc variants carrying lysine residues on the HBc VLP spikes. Utilising the introduced lysine residues and the intrinsic aspartic and glutamic acid residues exposed on the tips of the HBc spikes for chemical coupling of the chosen peptide and/or nucleic acid sequences ensured a standard and easy protocol for the further development of versatile HBc VLP-based vaccine and gene therapy applications.

Hepatitis B virus (HBV) core (HBc) protein p21, which is encoded by HBV gene C, acts in the viral life cycle as an icosahedral scaffold of the HBV nucleocapsid, which contains and carries genomic HBV DNA, polymerase (for a review, see^1^) and possibly protein kinase^2^. HBc protein spontaneously forms dimeric units^3^ that self-assemble into two particle isomorphs^2^,^4^ by allosterically controlled mechanisms^5^ in HBV-infected eukaryotic cells. The spatial structure of HBc particles was previously resolved in *E. coli* due to the ability of HBc to undergo synthesis and self-assembly in these cells (for a review see^6^). Recombinant HBc particles are represented by the same two isomorphs with triangulation numbers T = 4 and T = 3^7^; they consist of 240 and 180 HBc monomers and are 35 and 32 nm in diameter^7^,^8^, respectively. The three-dimensional structure of the T = 4 particles was resolved by X-ray crystallography^9^, whereas a quasi-atomic pattern of the native T = 3 isomorph was reconstructed by docking the dimers of the T = 4 crystal structure^8^.

HBc protein was also shown to self-assemble in multiple other efficient heterologous expression systems, including yeast *S. cerevisiae*^10^,^11^ and *P. pastoris*^12^,^13^. The HBc protein linear structure splits into the following two clearly separated domains: the N-terminal self-assembly (SA) domain (1–140 aa) that is necessary and sufficient to perform the assembly function and the protamine-like arginine-rich C-terminal domain (CTD; 150-183 aa)^14^. These two domains are separated by a hinge peptide (141–149 aa)^15^ that performs morphogenic functions and manages encapsidated nucleic acids^15^,^16^. The SA domain of HBc protein possesses a set of variable and conservative stretches that correspond to immunological B-cell epitopes and structural elements, respectively, whereas the CTD domain and hinge peptide are the most conserved HBc regions (for a review see^6^,^17^).

The intrinsic self-assembly function of the SA domain and its high capacity to accept foreign aa stretches are used to generate chimeric virus-like particles (VLPs), both full-length and C-terminally truncated, on the HBc scaffold (for review, see^6^,^18^,^19^,^20^). The CTD domain is responsible for the encapsidation of the 3.5-kilobase pregenomic HBV mRNA, which is converted into partially double-stranded HBV DNA^21^. Nucleic acid-binding sites in the CTD domain are located in four arginine blocks^22^. As a rather flexible structure, without any distinct tertiary outfit (although no 3D data are currently available), the CTD domain may appear inside^23^ as well outside of HBc particles^24^,^25^. According to recent findings, a significant portion of the CTD is exposed at the surface of the RNA-containing immature nucleocapsid, and the CTD is mostly confined within the DNA-containing mature nucleocapsid^26^.

Similar to natural HBc within viral nucleocapsids during the recognition of pregenomic HBV mRNA^21^, recombinant VLPs prefer single-stranded RNA for packaging, whereas the elimination of the CTD domain prevents this type of packaging in *E. coli* cells^27^,^28^. Nevertheless, HBc possesses a definite ability to bind to both RNA and DNA *in vitro*^22^; however, dsDNA is regarded as a poor substrate for assembly^29^.

Encapsidation of short hairpin RNAs by HBc nucleocapsids is performed *in vivo* in eukaryotic cells and results in the construction of the HBV “Trojan horse” vector that targets hepatocytes^30^. *In vitro* HBc encapsidation is regarded as a way to perform packaging of desired molecules, such as immunostimulatory (ISS) CpG sequences^31^,^32^ and other short oligodeoxynucleotides (ODNs)^33^, pregenomic mRNA and random ssRNA with similar efficiency irrespective of phosphorylation^34^,^35^. In addition, the packaging of ssDNA occurs to a lesser extent, and the packaging of dsDNA^34^, other polyanions (poly-glutamic acid and polyacrylic acid but not low molecular mass anions (inositol triphosphate) or polycations (polylysine and polyethylenimine)^34^, and magnetic nanoparticles^36^ occurs minimally.

However, the controlled encapsidation and quality control of bacteria- and yeast-derived HBc VLPs are hindered by the presence of irregular internal RNA of host origin. In this study, we propose a novel experimental approach to completely remove the internal RNA from bacteria- and yeast-derived HBc VLPs by alkaline hydrolysis, without any loss of VLP quality. The empty HBc VLPs demonstrated the high efficiency of simple, so-called contact DNA and RNA packaging. The introduction of lysine residues on the surface of HBc VLPs enabled chemical coupling of foreign peptide and nucleic acid sequences and promoted the development of packaging and peptide exposure technologies to generate well-characterised HBc-derived vaccine and gene therapy tools^37^,^38^.

## Results

### Preparation of empty HBc VLPs by alkaline treatment

**Figure 1:**
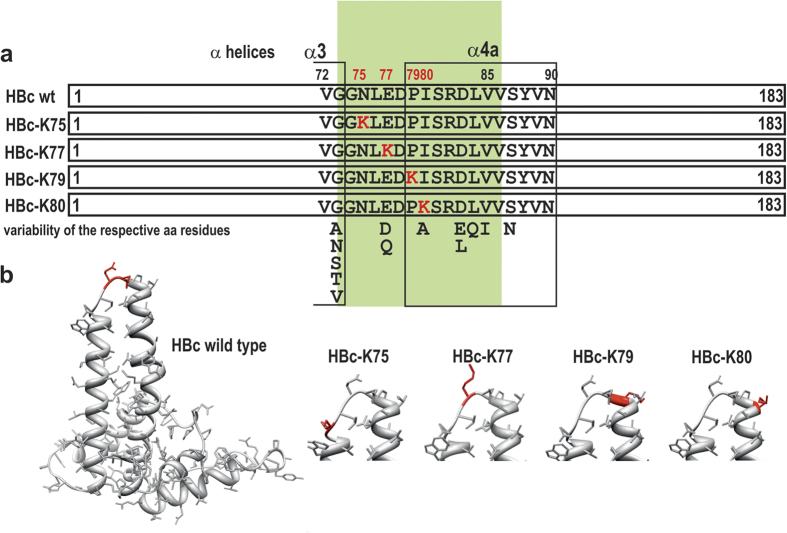
General structure of the initial wt HBc molecule from the HBV320 genome, genotype D1, subtype ayw2, GenBank accession number X02496^75^ and four variants exposing lysine residues at the tips of the spikes. (**a**) Primary structure of the central part of the HBc molecule, with alternative naturally occurring aa residues^6^,^17^. (**b**) 3D maps for the initial HBc monomer with Glu77 and Asp78 marked red and for the tips of the spikes of four lysine-exposing HBc variants with inserted lysine residues marked red. The maps are based on the crystal structure of recombinant HBc VLPs produced in bacteria^9^.

Wild type (wt) HBc VLPs were produced in *E. coli* and *P. pastoris.* Four HBc VLP variants with amino acid (aa) positions 75, 77, 79, and 80 substituted by lysine residues (Fig. 1), in order to expose the lysines on the tips of the HBc spikes for further chemical coupling purposes, were produced in *E.coli.*

Efficient column chromatography purification steps before alkaline treatment (see Supplementary Fig. S1 online) led to VLP preparations that demonstrated high consistency but different mobility by native agarose gel electrophoresis (NAGE) analysis at pH 8.3 and 7.5 (see Supplementary Fig. S2 online). Calculations (see Supplementary Methods online) based on precise UV spectra measurements (see Supplementary Fig. S3 online) revealed the total amount of VLP-encapsidated RNA as approximately 3844 and 3208 nucleotides per particle for bacteria- and yeast-produced wt HBc VLPs, respectively.

Length analysis of RNA encapsidated by the wt HBc VLPs from bacteria revealed the presence of fragments ranging from approximately 30 to 2000 nt (mononucleotides) in size, with the prevalence of short oligoribonucleotides up to 500 nt in length (see Supplementary Fig. S4 online).

Sequence analysis of RNA encapsidated by the bacteria-produced wt HBc VLPs showed that 34.93% of the RNA was represented by transcripts of the expression plasmid, 98.49% of which were mRNAs encoding the HBc monomer from the pHBc183 plasmid (see Supplementary Fig. S5 and Table S1 online). Other encapsidated sequences were of *E. coli* origin and represented at least 332 genes (see Supplementary MS Excel spreadsheet online). Remarkably, most of the VLP-packaged RNAs were of mRNA origin; 23S and 16S rRNA-derived sequences constituted only 5.82 and 0.61% of the total, respectively.

**Figure 2:**
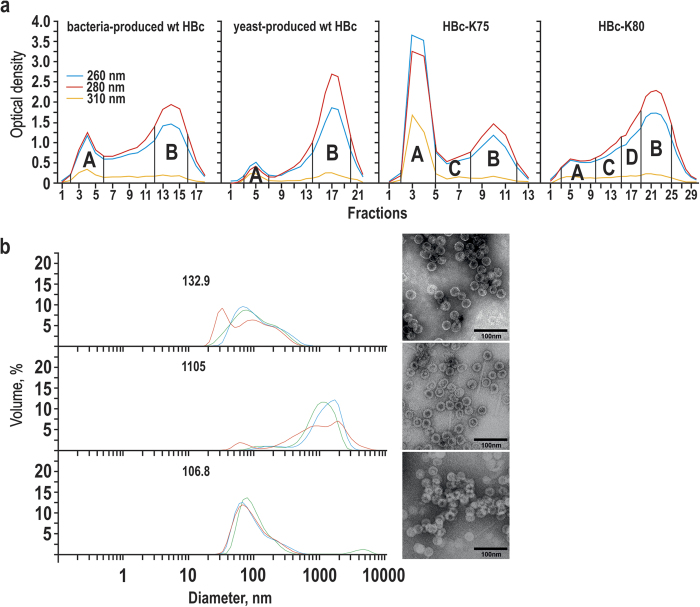
Separation of alkaline-treated HBc VLPs by Sepharose CL-4B column chromatography . (**a**) Chromatography of bacteria- and yeast-produced wt HBc VLPs as well as two representative lysine-exposing HBc VLP variants: HBc-K75 and HBc-K80. Pooled fractions are marked by capital letters. (**b**) DLS (left) and EM (right) characterisation of the fractions A of the alkaline-treated HBc VLP variants: bacteria-produced wt HBc (top), HBc-K77 (middle), HBc-K79 (bottom). Three independent DLS measurements are shown, mean particle diameters are indicated by numbers on the respective DLS graphs.

RNA encapsidated by wt and four lysine-exposing HBc VLPs was fully hydrolysed by alkaline treatment, and characteristic VLP fractions were pooled after separation on Sepharose CL-4B (Fig. 2a). PAGE showed the maintenance of HBc during alkaline treatment (see Supplementary Fig. S1 online). The A fractions of the HBc VLPs corresponded by elution time to VLP aggregates and demonstrated contamination with RNA by UV spectra analysis (see Supplementary Fig. S6 online). Dynamic light scattering (DLS) measurements confirmed marked aggregation of VLPs, with the highest level of aggregation for HBc-K77 VLPs (Fig. 2b, left side). Nevertheless, EM of the A fractions demonstrated a rather standard VLP pattern (Fig. 2b, right side).

The B fractions of the HBc VLPs corresponded to the expected elution time from the column based on the VLP molecular mass and demonstrated UV spectra typical for proteins without any remarkable RNA contamination (see Supplementary Fig. S6 online). The B fraction prevailed over A fraction in bacteria- and yeast-produced wt HBc VLPs (Fig. 2a). However, four lysine-exposing HBc VLP variants demonstrated two different profiles characterised by the (i) prevailing A fraction and a clear disposition to aggregation in the case of HBc-K75 and HBc-K77 and (ii) prevailing B fraction and low level of aggregation in the case of HBc-K79 and HBc-K80. For this reason, only one representative chromatography pattern for each of the two groups, namely, HBc-K75 or HBc-K80, is shown in Fig. 2a. In contrast, Fig. 2b depicts two other representatives of the two lysine-exposing HBc VLP groups (HBc-K77 and HBc-K79). Yield of the B fraction in case of the bacteria-produced wt VLPs was 4.6 mg/g cells, which corresponded to 60% of material obtained after alkaline treatment and before fractionation. Approximately the same yield was observed for yeast-produced wt HBc VLPs. Regarding the lysine-exposing HBc VLPs, a higher yield of the B fraction was observed for HBc-K79 and HBc-K80 VLPs of approximately 2.8 mg/g cells, whereas the other two variants yielded only about 0.8 mg/g cells.

**Figure 3:**
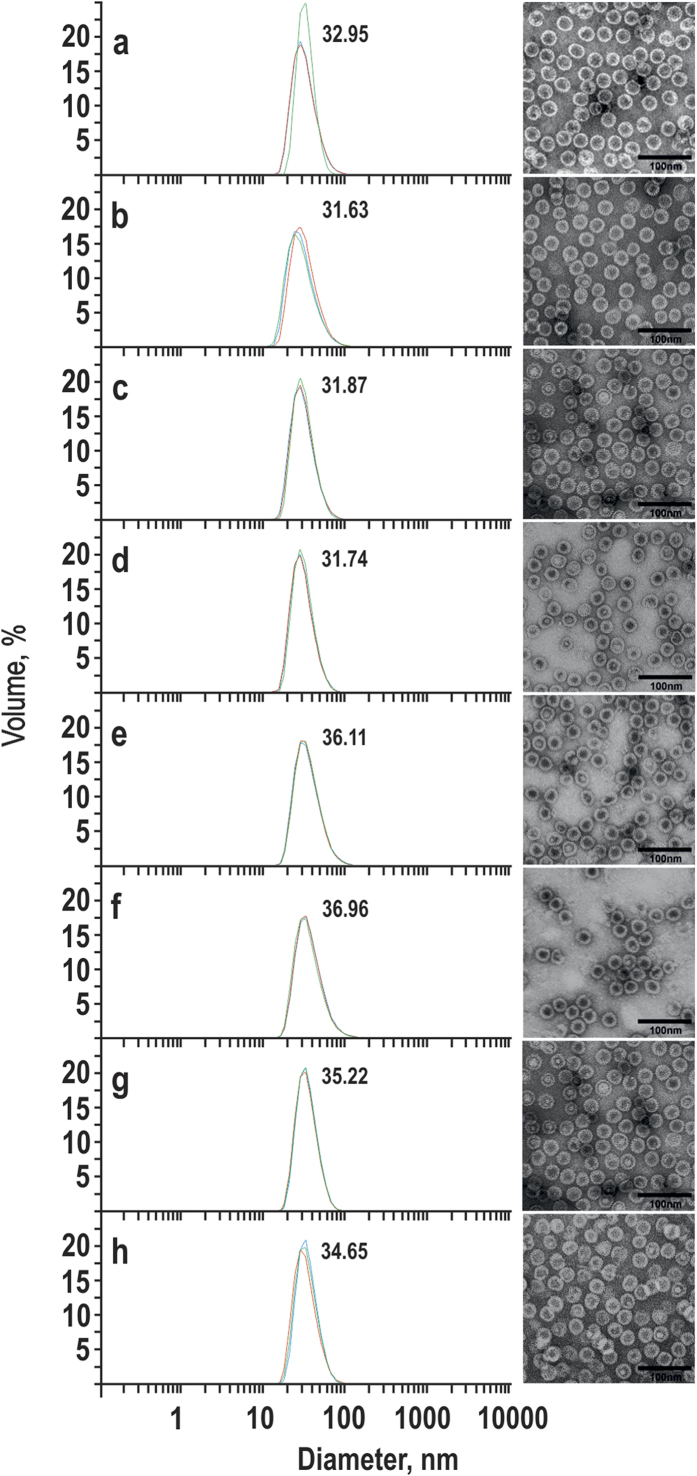
DLS (left) and EM (right) characterisation of the fractions B of the alkaline-treated HBc VLP variants . (**a**) Bacteria-produced wt HBc, (**c**) yeast-produced HBc, (**e**) HBc-K75, (**f**) HBc-K77, (**g**) HBc-K79, (h) HBc-K80. Bacteria- and yeast-produced wt HBc VLPs before alkaline treatment are shown for a comparison in (**b**) and (**d**), respectively. Three independent DLS measurements are shown, mean particle diameters are indicated by numbers on the DLS graphs.

The empty HBc VLPs of B fractions demonstrated up to 100% size homogeneity by DLS analysis and high quality electron micrographs (Fig. 3). No significant DLS or EM differences were found by comparing the empty HBc VLPs with “natural” bacteria- or yeast-produced wt HBc VLPs before alkaline treatment (Fig. 3, rows b and d). DLS diameter measurements of both “natural” and alkaline-treated wt HBc VLPs from bacteria and yeast indicated interval approximately 31–33 nm in size (Fig. 3, rows a–d), whereas the lysine-exposing HBc variants appeared a bit larger, namely, 35–37 nm (Fig. 3, rows e-h).

As shown in Fig. 2a, the HBc VLPs from the intermediate-pooled chromatography fractions C and D demonstrated a lower level of aggregation than VLP products from fractions A by DLS analysis, and no evident signs of aggregation were shown by EM (for typical examples see Supplementary Fig. S7 online).

**Figure 4:**
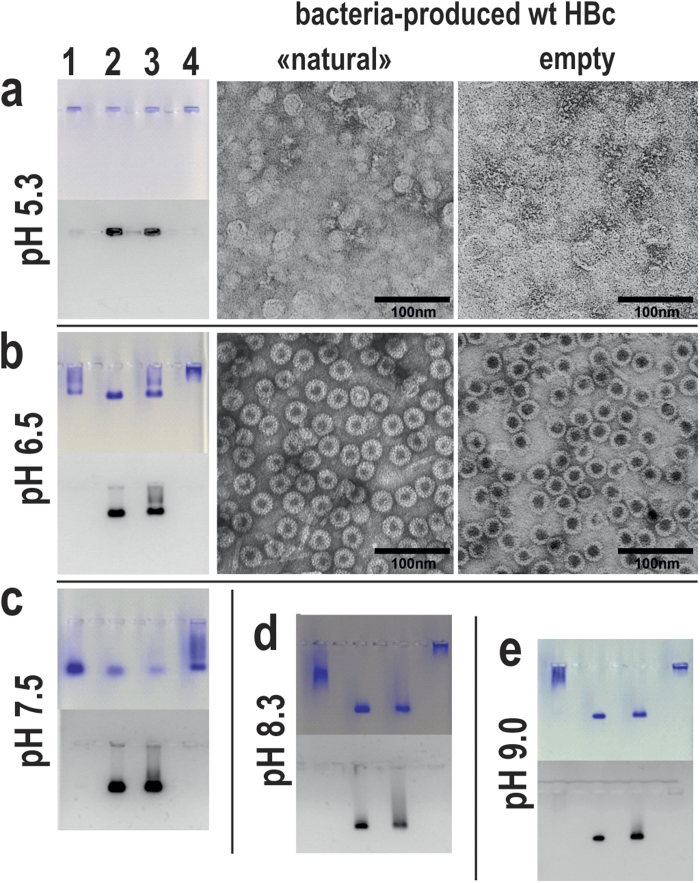
Comparison of “natural” and empty wt HBc VLPs by NAGE (left) and EM (right) at different pH values. Tracks correspond to the following wt HBc VLP preparations: yeast-produced empty (1) or “natural” (2), bacteria-produced “natural” (3) or empty (4). Gels are stained by Coomassie (upper part) and ethidium bromide (lower part).

NAGE revealed the destruction of both traditionally purified (“natural”) and empty HBc VLPs in acidic conditions (Fig. 4, row a). Starting from pH 6.5 until basic conditions at pH 12, “natural” and empty VLPs demonstrated standard EM characteristics (Fig. 4, row b). VLP mobility in NAGE was dependent on the surface charge of VLPs at the specific pH value. The mobility of initial RNA-filled and empty particles was similar at pH 7.5 (Fig. 4, row c). The CTD positive charge reached its maximum at pH 9.0, resulting in a total neutral charge of empty particles, which prevented the movement of empty HBc VLPs in NAGE (Fig. 4, row e). Remarkably, bacteria- and yeast-derived empty HBc VLPs demonstrated different mobility at pH 8.3 (Fig. 4, row d), which may be explained by the presence of the exposed phosphate group at phosphorylated aa position Ser87 in the case of yeast-derived VLPs^13^.

The lysine-exposing HBc variants moved slower than both wt HBc VLP variants because of the additional positive charge on the surface. Remarkably, the empty lysine-exposing VLPs preserved the same mobility characteristics in NAGE as their “natural” counterparts at pH 7.5. As an exception, both empty and “natural” HBc-K77 VLPs remained at the starting position in the NAGE at pH 7.5 and pH 8.3, which was most likely due to the neutralising effect of Glu78 on the neighbouring Lys77 residue (see Supplementary Fig. S2 online).

Antigenicity of HBc VLPs was characterized by (1) two standard commercial Siemens Enzygnost monoclonal kits: HBe/anti-HBe and HBc/anti-HBc, (2) in-house Ouchterlony’s double radial immune diffusion test with (i) polyclonal rabbit antibodies or (ii) monoclonal C1-5 antibody recognizing 78-DPIxxD-83 epitope^39^ as counter reagents. No differences in behaviour of empty and “natural” HBc VLP variants were found. All HBc VLP variants did not react in the Enzygnost HBe/anti-HBe test and confirmed therefore high self-assembled integrity of empty VLP preparations. In contrast to bacteria- and yeast-produced wt HBc, as well as HBc-K75 and HBc-K79 VLPs, the HBc-K77 and HBc-K80 VLPs were not detectable by the Enzygnost HBc/anti-HBc kit. All HBc VLP variants formed precipitation lines in the Ouchterlony immune diffusion test using polyclonal rabbit anti-HBc antibodies, but not monoclonal C1-5 antibody, which was unable to precipitate HBc-K80 VLPs (see Supplementary Fig. S8 online).

### Encapsidation of ribonucleic acid by empty HBc VLPs

**Figure 5:**
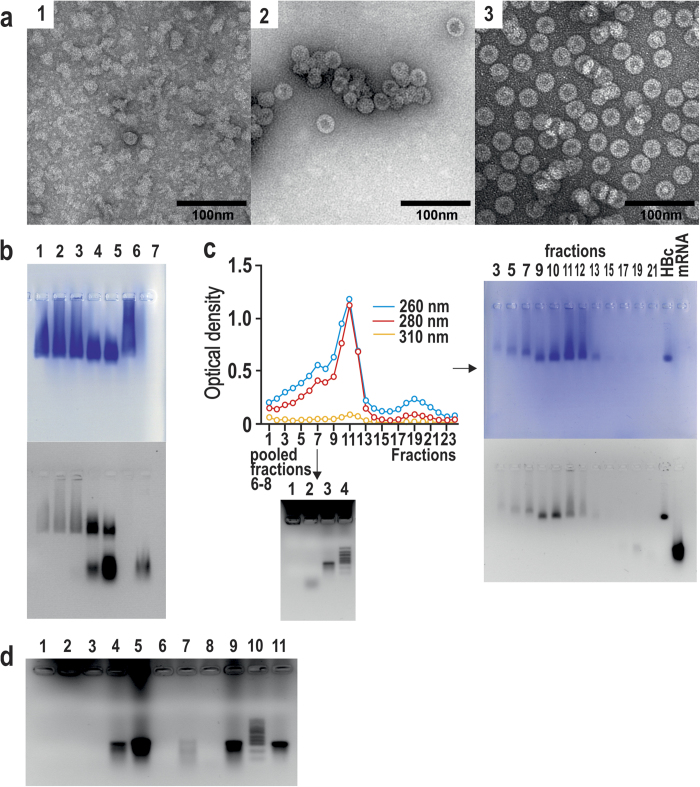
Restoration of empty bacteria-produced wt HBc VLPs after urea treatment or direct contact by RNA packaging. (**a**) Electron micrographs of HBc VLPs after 7 M urea treatment (1) and restoration of HBc VLPs without any additional RNA (2) or by *E. coli* ribosomal RNA predominance (8000 mononucleotide per one VLP) (3). (**b**) NAGE analysis by Coomassie (top) and ethidium bromide (bottom) staining of the DTA mRNA contact encapsidation by empty yeast-produced wt HBc VLPs at molar VLP/mRNA ratios 1:0.23 (1), 1:0.34 (2), 1:0.45 (3), 1:0.91 (4), 1:1.81 (5), and empty yeast wt HBc VLPs (6) and DTA mRNA (7) as controls. (**c**) Sucrose gradient centrifugation of DTA mRNA-packaged yeast-produced empty wt HBc VLPs with NAGE analysis of fractions (Coomassie (top) and ethidium bromide (bottom) staining) and formaldehyde agarose gel electrophoresis (FAGE) of pooled VLP fractions 6-8 before (1) and after (2) phenol treatment, DTA mRNA (3) and RNA ladder (4) as controls. (**d**) Fate of DTA mRNA within the packaged bacteria- and yeast-produced wt HBc as well as HBc-K75 VLPs by FAGE analysis. Phenol-treated samples of the bacteria- (1–4) and yeast- (6–9) produced empty wt HBc VLPs contacted with DTA mRNA at VLP versus mRNA molar ratio 1:0.85 (2 and 7), empty wt HBc VLPs saturated with DTA mRNA and purified by CsCl centrifugation (3 and 8), the same with the addition of 0.85 molar proportion of non-packaged DTA mRNA (4 and 9); empty wt HBc VLPs (1 and 6) and DTA mRNA (5), as well as phenol non-treated RNA ladder (10) and DTA mRNA (11) were taken as controls.

Bacteria- and yeast-produced empty wt HBc VLPs and the four lysine-exposing VLP variants were loosened in 7 M urea. The electron micrographs of the urea-treated destroyed (Fig. 5a, micrograph 1) and fully restored particles: after removal of urea by dialysis without any additions (Fig. 5a, micrograph 2) or in the presence of added bacterial rRNA (Fig. 5a, micrograph 3) are shown for the bacteria-produced wt HBc VLPs. Further RNA encapsidation experiments showed that all studied HBc VLP variants packaged RNA immediately after direct contact of RNA and VLPs without the urea treatment step.

Preliminary quantification of RNA packaging was performed by titrating all of the studied VLPs using *E. coli* tRNA (see Supplementary Fig. S9 online for the packaging of bacteria- and yeast-produced wt HBc VLPs as examples) and rRNA (see Supplementary Fig. S10 online for the packaging of HBc-K75 as an example) with further NAGE analysis. The NAGE bands corresponding to the packaged VLPs are depicted by Coomassie and ethidium bromide staining and demonstrate higher mobility than their empty counterparts. The packaging capacity of empty bacteria-produced wt HBc VLPs was approximately two-fold higher than the same for the yeast-produced analogues. A part of the added tRNA appeared as unbound material in the case of yeast-produced wt HBc by 10-fold molar excess of tRNA; however, in the case of bacteria-produced wt HBc, the 25-fold molar tRNA excess was necessary to leave unbound tRNA (see Supplementary Fig. S9 online). The encapsidated RNA remained fixed to the VLPs after gel filtration (see Supplementary Fig. S9 online for the tRNA packaging as an example) and ammonium sulphate precipitation. Packaged HBc VLPs did not differ from “natural” or empty HBc VLPs by their DLS-measured diameters (see Supplementary Fig. S10 online for the HBc-K75-performed rRNA packaging as an example). Phenol extraction of the VLP-packaged RNA demonstrated strong tRNA (see Supplementary Fig. S9 online) and rRNA (see Supplementary Fig. S11 online) degradation as determined by PAGE and the BioAnalyzer, respectively.

A representative study of RNA incorporation into HBc VLPs by direct contact was performed for the encapsidation of well-characterised purified 1221 nt-long diphtheria toxin fragment A (DTA) mRNA (see Supplementary Fig. S12 and Supplementary Protocols online) as shown in Figs 5b–d. Figure 5b depicts a titration example of yeast-produced wt HBc VLPs with DTA mRNA that revealed optimal VLP/mRNA molar ratios of not more than one mRNA per one HBc particle. Figure 5c shows the stable retention of encapsidated mRNA within the VLPs during sucrose gradient centrifugation. The encapsidated DTA mRNA remained within particles during Sepharose CL-2B column chromatography and sedimentation with ammonium sulphate (see Supplementary Fig. S13 online). Empty bacteria-produced HBc VLPs bound more mRNA than yeast-produced empty VLPs. Specifically, in equal reaction mixtures, more mRNA remained unbound when incubated with empty yeast-produced HBc VLPs (see Supplementary Fig. S13 online).

**Figure 6:**
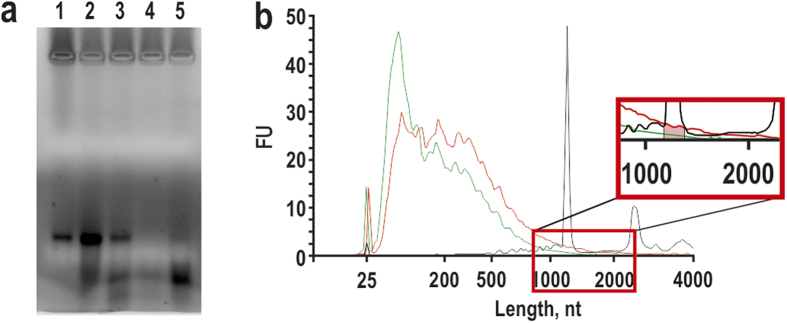
Length distribution of HBc VLP-encapsidated DTA mRNA with the BioAnalyzer 2100. (**a**) DTA mRNA after purification through the oligo-dT cellulose column before (1) and after (2) phenol/chloroform purification (lane (2) has two-fold more mRNA than lane (1)); extracted content from contact packaging experiments of DTA mRNA and bacteria-produced HBc VLPs (3) or empty bacteria-produced HBc VLPs (4) (in molar ratios 1:2); lane (5) shows extracted content from contact incubation of bacteria-produced HBc VLPs with DTA mRNA, that purified through a Sepharose CL-2B column chromatography where fraction containing non-aggregated VLPs and lacking free RNA were taken. (**b**) mRNA before packaging (black) and after extraction from bacteria- (red) or yeast- (green) produced wt HBc VLPs.

The encapsidated RNA material differed markedly from the initial mRNA by length and demonstrated clear degradation features in formaldehyde agarose gel electrophoresis (FAGE) (Fig. 5d). Furthermore, fresh extra portions of unpackaged DTA mRNA that were added to mRNA-packaged HBc VLPs remained stable and did not demonstrate any signs of mRNA degradation (Fig. 5d). In contrast to empty particles incubated with DTA mRNA, naturally packaged HBc VLPs displayed no mRNA degradation (Fig. 6a). The percentage of the RNA material recovered from purified HBc VLPs and DTA mRNA complexes was approximately 13% and 19% for bacteria- and yeast-produced HBc VLPs, respectively. The percentage of the RNA material recovery was obtained by comparison of ethanol precipitated amount (after phenol/chloroform extraction) with theoretically calculated packaged amount. In comparison, the technical recovery of mRNA itself was approximately 50%. To estimate the consistency and fate of the packaged DTA mRNA, sequencing of the unpacked mRNA material was performed by massive parallel sequencing using the Ion Torrent PGM technique according to a protocol described in the Supplementary Methods section. DNA libraries were prepared for the exhaustive sequencing of VLP-packaged nucleic acid material. According to the sequencing data, more than 98% of the material before packaging was consistent with DTA mRNA (see Supplementary Table S1 online). Figure 6b presents a typical example of the length distribution of the nucleic acid extracted from bacteria- or yeast-produced wt HBc VLPs in comparison with DTA mRNA before packaging. Only approximately 1.5% of the unpacked material may be represented by full-length DTA mRNA (see inset in Fig. 6b). Just the same is shown in FAGE (Fig. 6a, lane 5) where the RNA material from the bacteria-produced wt HBc VLPs packaged with DTA mRNA was analysed. Most of the RNA material appeared as degraded and only a weak band of the full-length DTA mRNA fragment was observed. The profiles of respective DNA libraries that were created for the sequencing of extracted RNAs (see Supplementary Fig. S14 online) confirmed the mRNA degradation. Regarding HBc-K80 VLPs, analysis of the internal content showed that 86.77% of the sequences were DTA mRNA-derived, only 1.64% were pHBc183-derived and 11.59% were *E. coli*-derived (see Supplementary Fig. S15 and Table S1 online).

### Encapsidation of deoxyribonucleic acid by empty HBc VLPs

Encapsidation of the following three representative DNA categories: (i) relatively short single-stranded oligodeoxynucleotides (CpG ODNs), (ii) full-length plasmids, and (iii) long double-stranded DNA fragments (for the full list of the DNA structures used for the DNA encapsidation studies see Supplementary Table S2 online) by different empty HBc VLPs occurred with similar efficacy either via VLP restoration after 7 M urea treatment or via direct contact of empty VLPs with DNA samples.

**Figure 7:**
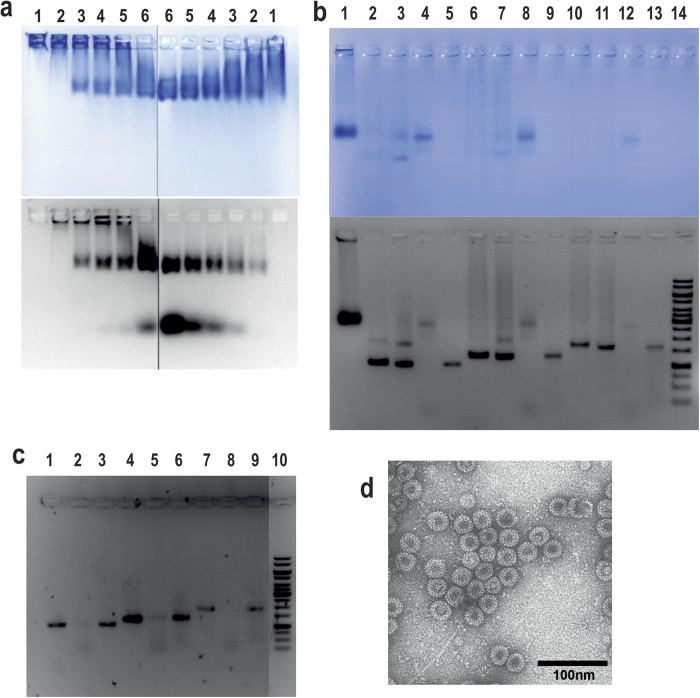
Packaging of bacteria- and yeast-produced wt HBc VLPs by a DNA fragment and two CpG ODNs. (**a**) Contact titration of bacteria-produced (left part) and yeast-produced (right part) wt HBc VLPs by ODN63 at appropriate VLP molar superiority over ODN: 10 (2), 20 (3), 30 (4), 40 (5), 50 (6) and the appropriate empty HBc VLPs as a control (1). (**b**) NAGE analysis of restoration of bacteria-produced wt HBc VLPs by DNA fragments of 1047, 1289, and 1737 bp in length. Coomassie- (top) and ethidium bromide- (bottom) stained gels of the restored encapsidated HBc VLPs (all purified by CsCl density gradient centrifugation) at the fragment to HBc VLP molar ratio 1:1.5 in the case of the fragments 1047 bp (2–5), 1289 bp (6–9), and 1737 (10–12) bp where samples (2,6,10) are in a restoration buffer, (3,7,11) are in DNase buffer, and (4,8,12) are samples in DNAse buffer and treated by DNase before phenol extraction; controls: 100 bp Plus DNA ladder (1), the respective DNA fragments (5,9,13), initial alkaline non-treated bacteria-produced wt HBc VLPs (14). (**c**) Ethidium bromide-stained NAGE of phenol-extracted content of the restored HBc VLPs carrying fragments 1047 (1–3), 1289 (4–6), and 1737 (7–9) bp where the encapsidated VLPs were not treated (1,4,7) or treated with DNase (2,5,8) before phenol extraction; controls: the respective DNA fragments (3,6,9), 1 kb ladder (10). (**d**) EM of bacteria-produced wt HBc VLPs restored with 1289 bp DNA fragment and treated with DNAse.

First, VLP titration by short 20 nt single-stranded CpG ODNs (see Supplementary Fig. S16 online) or triplicate (63 nt) CpG ODNs (Fig. 7a) was performed. High ODN excess over HBc VLPs led to the optimal encapsidation of both ODN forms. Technical documentation of the ODN63 encapsidation process by bacteria- and yeast-produced wt HBc VLPs is presented in Table S3 (see Supplement online). Overall, empty bacteria-produced wt HBc VLPs demonstrated a higher capacity of packaging than the empty yeast-produced wt HBc VLPs. The complexes of HBc VLPs and ODNs retained their stability during NAGE and sucrose and CsCl density centrifugation (see Supplementary Fig. S17 online).

**Figure 8:**
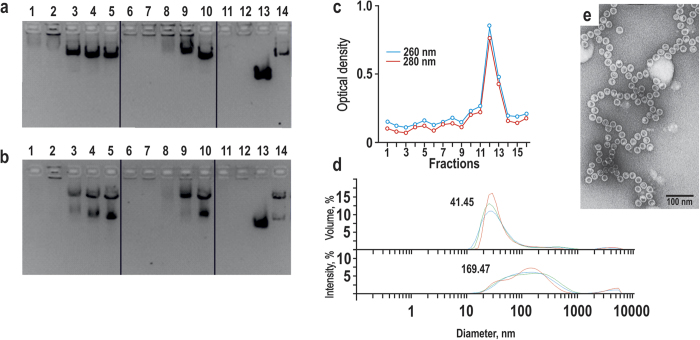
NAGE analysis of contact encapsidation of the pCEP-CXCR4-eGFP plasmid by empty bacteria- and yeast-produced HBc VLPs. (**a**) Titration of yeast-produced (1–5) and bacteria-produced (6–10) HBc VLPs (148 pmol) by varying pCEP-CXCR4-eGFP amounts: 52 pmol (1), 26 pmol (2), 13 pmol (3), 7 pmol (4), 3 pmol (5) with ratios indicated in Table S4 (see Supplementary online). Controls: empty yeast-produced HBc VLPs (11), empty bacteria-produced wt HBc VLPs (12), initial bacteria-produced alkali non-treated HBc VLPs (13), pCEP-CXCR4-eGFP plasmid (14). (**b**) Titration of yeast-produced (1–5) and bacteria-produced (6–10) HBc VLPs (74 pmol) by varying amounts of the plasmid pCEP-CXCR4-eGFP SalI fragments: 52 pmol (1), 26 pmol (2), 13 pmol (3), 7 pmol (4), 3 pmol (5) with the same ratios as indicated in Table S4 (see Supplementary online) for pCEP-CXCR4-eGFP plasmid. Controls: empty yeast-produced HBc VLPs (11), empty bacteria-produced wt HBc VLPs (12), initial bacteria-produced alkali non-treated HBc VLPs (13), pCEP-CXCR4-eGFP plasmid cleaved by SalI (14). (**c**) Yeast-produced wt HBc VLPs restored by pCEP-CXCR4-eGFP plasmid in 53-fold molar ratio of VLP versus plasmid CsCl density gradient centrifugation. (**d**) DLS of the complex of bacteria-produced empty HBc VLPs restored by 10-fold molar excess of VLPs over pCEP-CXCR4-eGFP plasmid after CsCl density gradient centrifugation, presented by volume (top) or intensity (bottom) measurements. (**e**) Electron micrograph of the complex formed by contact of empty yeast-produced wt HBc VLPs and pCEP-CXCR4-eGFP plasmid (ratio 53:1) after Sepharose CL-2B gel chromatography.

The second round of the encapsidation experiments included contacting of empty VLPs with long circular or linearised plasmids or plasmids restricted by rarely-cleaving restriction enzymes producing, for example, fragments of 7871 and 12268 bp (base pairs) in length. These encapsidations were performed in high excess of HBc VLPs (up to 50-fold molar excess over DNA) and resulted in the formation of complexes remaining in the start pockets of NAGE; such complexes were stable during CsCl density gradient centrifugation (see Fig. 8 and Supplementary Table S4 online) and size exclusion column chromatography (see Supplementary Fig. S18 online).

More details on the structure of the involved plasmids and the stability of the HBc VLP-DNA complexes are presented in the Supplement (see Supplementary Fig. S19 and Fig. S20 online). DLS measurements revealed marked VLP aggregation as demonstrated by the intensity analysis mode when compared with the volume mode (Fig. 8d). EM analysis demonstrated the presence of VLP chains (Fig. 8e) that may have been the result of VLP attachment to long DNA molecules. Plasmids and large DNA fragments in such complexes were protected by HBc VLPs against DNase cleavage (Fig. 8a,c).

The third round of the encapsidation experiments was performed with individual DNA fragments of different lengths at approximately equimolar ratios of VLPs to DNA. For example, titration performed with different amounts of VLPs by a relatively short DNA fragment of 601 bp showed that the fragment is packaged into individual particles and not to VLP chains (see Supplementary Fig. S16 online). Next, the maximal size of the packaged DNA fragment that after proper packaging was protected against DNase cleavage was established. Phenol elution of HBc VLP-packaged DNA after DNase treatment of HBc VLP-DNA complexes showed that DNA fragments of 1047 bp (see Supplementary Fig. S21 online) and 1289 bp (Figs 7b–d) were protected; however, 1811 bp (see Supplementary Fig. S21 online) and 1737 bp (Figs 7b–d) fragments were not protected against DNase cleavage. Therefore, the border of the HBc VLP encapsidation-allowed length of double-stranded DNA fragments is located between 1289 and 1737 bp. The quality of the HBc VLPs carrying a 1047 bp DNA fragment after DNase treatment was assessed by DLS measurements (see Supplementary Fig. S22 online). HBc VLPs carrying a 1289 bp DNA fragment after DNase treatment showed intact particles by EM (Fig. 7d).

## Discussion

HBc particles are complicated multifunctional nanodevices that perform numerous functions in strict order during HBV replication, such as successive dissociation/re-association^40^ and phosphorylation/dephosphorylation^41^,^42^,^43^,^44^,^45^. These processes are necessary for nuclear targeting and entry, pregenomic mRNA encapsidation, dsDNA synthesis, nucleocapsid maturation, envelopment, and budding and release (for review see^1^,^46^,^47^).

Due to the highly efficient expression of HBc and its chimeric derivatives in *E. coli* (for review see^18^,^19^,^20^), HBc remains a widely used VLP carrier for the generation of putative vaccines over the past 28 years^48^,^49^. In addition to traditional vaccine applications, chimeric HBc VLPs have been suggested as candidate tools for specific cell targeting^50^. In addition to classical methods to expose foreign aa stretches to HBc spikes, various advanced approaches, such as SplitCore^51^,^52^, nano-glue^53^, and metalloporphyrin complexes on hexahistidine tags, have been recently elaborated^54^.

HBc applications are connected with its ability to recognise HBV nucleic acids, in which HBc phosphorylation is necessary and sufficient for pregenomic RNA encapsidation^55^ and HBc dephosphorylation enables genomic dsDNA synthesis^56^ and triggers maturation (i.e., envelopment and secretion)^44^,^57^. Mature DNA-containing HBc particles demonstrate significant differences in structure versus immature RNA-containing particles^58^. DNA appears as a poor substrate for encapsidation; therefore, dsDNA-filled HBc particles are spring-loaded^59^. Moreover, a significant portion of the CTD is exposed at the surface of the immature RNA-containing HBc particles, whereas the CTD is mostly confined within mature DNA-containing HBc particles^26^.

Recombinant HBc particles purified from *E. coli* cells contain heterologous RNA in amounts comparable with the pregenomic HBV mRNA content^35^,^59^,^60^. In accordance with our results, yeast-produced HBc VLPs reveal packaging of approximately 3000–3200 nt. The bacteria-produced HBc VLPs demonstrate a higher level of packaging by at least 300 nt, which appears to correlate with at least 240 phosphogroups on the inner HBc VLP surface as a result of HBc protein phosphorylation in yeast^13^.

Traditional attempts to prepare full-length HBc particles for *in vitro* packaging by desired molecules are based on the following two approaches: (i) non-dissociating osmotic shock^61^ and (ii) full HBc dissociation after micrococcal nuclease^34^ or guanidine chloride^35^ treatment. Interestingly, incorporated RNA can be removed by a nuclease, which is covalently added to the HBc protein and displayed on the inner surface of HBc VLPs^62^. Regarding practical packaging of functionally important material, only successful encapsidation of short ISS CpG ODNs has been previously achieved^31^,^32^,^63^,^64^.

Here, we propose a technological solution that combines both epitope exposure and nucleic acid packaging approaches, which promotes versatile HBc VLP applications in vaccinology and gene therapy. First, surface-exposed lysine residues that are applicable for chemical coupling of foreign peptides or oligonucleotides were introduced on the protruding HBc spikes. Natural HBc molecules harbour only two lysine residues at positions 7 and 96 that are positioned on the external VLP surface at the base of the HBc spikes. Therefore, the CTD positive charge is ensured by the arginine, while the lysine residues play a specific role in HBc ubiquitination^65^. Although lysine never appears at the selected positions in naturally mutated HBc variants^6^,^17^, the incorporation of lysine residues at the fully conserved positions 75 and 79 and minimally variable positions 77 and 80 (Fig. 1) does not prevent self-assembly. Second, we performed exhaustive purification and standardisation conditions of bacteria- and yeast-produced HBc VLPs by full deprivation of contaminating encapsidated RNAs during simple, rapid, and highly efficacious alkaline treatment at pH 12. This method also efficiently purified four HBc variants carrying lysine residues on the tips of the HBc spikes. Recently, efficiency of the alkaline treatment was demonstrated for purification of RNA phage PP7 VLPs carrying human papillomavirus epitopes^66^.

The obtained empty particles encapsidated both RNA and DNA by the following two alternate approaches: (i) the restoration of HBc VLPs after 7 M urea treatment and (ii) direct contact of nucleic acids with empty HBc VLPs. Notably, the encapsidation of bacterial rRNA and specific DTA mRNA was accompanied by the cleavage of the packaged RNA, and the excessive unpackaged RNA remained stable. This phenomenon suggests that HBc, itself, may possess ribonucleolytic activity that prevents the encapsidation of unspecific RNAs *in vivo*. Nucleoproteins, structural analogues of the HBc protein, possess exoribonuclease activity in the case of Lassa and Tacaribe viruses belonging to arenaviruses^67^,^68^ and endodeoxyribonuclease activity in the case of Crimean-Congo haemorrhagic fever virus belonging to bunyaviruses^69^.

The HBc VLPs may protect the long double-stranded DNAs that exceed the length of the HBV genome from DNase attack by forming VLP chains that cover the DNA. This protection is mediated by a strong excess of HBc VLPs compared with the DNA. When the VLP to DNA ratio is approximately equimolar, HBc VLPs package DNA fragments of approximately 1200 bp in length. The most efficient packaging was achieved for single-stranded CpG ODNs during the strong ODN excess compared with HBc VLPs.

Comparing bacteria- and yeast-produced HBc particles, no significant difference was observed in their ability to reassemble and package desired RNAs or DNAs. Both HBc particle products are technologically equal in semi-preparative propagation and purification trials. However, the encapsidation capacity of the bacteria-produced HBc VLPs is always higher (Fig. 7a) than that of the yeast-produced HBc VLPs, which is in full accordance with the above-mentioned differences in the initial RNA content of both purified particles and is connected with the phosphorylated status of yeast-produced HBc VLPs^13^.

Unusually high packaging capacity of HBc particles can be explained by their extended inner space due to the absence of bulky HBV-related proteins, such as the viral polymerase. Highly efficient and technologically sound packaging ranks the HBc carrier with other VLP candidates used for nucleic acid packaging, such as polyoma and papilloma viruses, RNA phages, and cowpea chlorotic mottle virus (for references see^20^).

Strong advantages of utilising the HBc carrier consist of the capacity to employ the proposed packaging technology in combination with traditional approaches to construct chimeric HBc VLPs as vaccines and/or cell targeting tools because HBc protein is one of the most studied VLP models (for review, see^18^,^19^,^20^). Furthermore, HBc particles possess a highly specific nuclear targeting ability, which may allow for the highly specific delivery of packaged DNA to the cell nucleus (for review, see^1^). Finally, initial HBc VLPs, without any added ligands, are naturally targeted to B lymphocytes because the latter function as antigen-presenting cells (APCs) both in murine^70^ and human^71^,^72^ B cells. Employing B cells as APCs for HBc protein explains its enhanced immunogenicity in mice and humans and the contribution of the HBc antigen in the induction and/or maintenance of chronic HBV infection^70^.

In summary, the proposed versatile approach is beneficial for vaccine production by exposing foreign epitopes through the chemical coupling of the integrated lysine residues, by the efficient packaging of CpG ODNs and by the potential immunostimulation of ribonucleic acids. Furthermore, this method also promises gene therapy applications by exposing cell targeting peptides and/or oligonucleotides and by the efficient packaging of relatively long DNA fragments.

## Methods

### Bacterial strains

*Escherichia coli* strain RR1 [F^−^_ r_B_^−^ m_B_^−^ *leuB6 proA2 thi-1 araC14 lacY1 galK2 xyl-5* *mtl-1 rpsL20* (Str^r^) *glnV44*_Δ(*mcrC-mrr*)] was used for cloning purposes. *E. coli* strain K802 (F^−^ r_K_^−^ m_K_^+^ *e14 McrA metB1lac Y1 [or lacI-Y6] galK2 galT22 glnV44 mcrB*) was used for the transformation of the plasmids expressing recombinant HBc proteins.

### Plasmid construction for HBc VLP expression

The lysine-exposing variants of HBc protein were constructed by standard mutagenesis procedures using the pHBc183 plasmid expressing HBc gene with codons optimised for synthesis in *E. coli*^60^. Four mutant plasmids were constructed: pHBc-K75 with primers pINC-312 (5´-GCTACCTGGGTGGGTGGTAAATTGGAAGATCCAATATC-3´) and pINC-313 (5´-GATATTGGATCTTCCAATTTACCACCCACCCAGGTAGC-3´); pHBc-K77 with primers pINC-314 (5´-GCTACCTGGGTGGGTGGTAATTTGAAGGATCCAATATC-3´) and

pINC-315 (5´-GATATTGGATCCTTCAAATTACCACCCACCCAGGTAGC-3´); pHBc-79K with primers pINC-572 (5´-GGTAATTTGGAAGATAAAATATCCAGGGACCTAGT-3´) and

pINC-573 (5´-ACTAGGTCCCTGGATATTTTATCTTCCAAATTACC-3´); and pHBc-80K with primers pINC-574 (5´-GGTAATTTGGAAGATCCAAAATCCAGGGACCTAGTAGT-3´) and pINC-575 (5´-ACTACTAGGTCCCTGGATTTTGGATCTTCCAAATTACC-3´).

### HBc VLP production

Regarding *E. coli*, after cell transformation with the appropriate plasmids, 5 ml of LB liquid medium supplemented with 20 μg/ml ampicillin was inoculated with a single colony and incubated at 37 °C for 16–24 h without shaking. The prepared inoculum was diluted 50-fold with 2xTY medium containing 20 μg/ml ampicillin and supplemented with 2 g glucose, 3.47 g KH_2_PO_4_, 18.8 g K_2_HPO_4_ per litre and incubated at 37 °C overnight on a shaker (200 rpm). The cells were harvested by centrifugation. Regarding *P. pastoris*, HBc VLP expression and purification were performed according to the protocol described in^13^.

### Deprivation of HBc particles from encapsidated RNA by alkaline treatment

To prepare the HBc VLPs for alkaline treatment, the purified particles were solubilised in 7 M urea, placed onto a Sephacryl S300 column (1.4 × 55 cm) and chromatographed by elution with 0.1 M Na_2_CO_3_, 2 mM DTT solution at a velocity of 2 ml/h (30 min/1 ml fraction). Pure fractions (see Supplementary Fig. S1 online) were transferred to a dialysis tubing cellulose membrane (Sigma Aldrich) and dialysed intensively against 100 ml of an “alkaline” solution (0.1 M Na_2_HPO_4_ / Na_3_PO_4_, 0.65 M NaCl, pH 12 (NaOH)) at 37 °C for 18 h. Then, the “alkaline” solution was exchanged for a restoration buffer (0.1 M Na_2_HPO_4_, 0.65 M NaCl, pH 7.8 (H_3_PO_4_)), and the material was dialysed at room temperature for 1 h and then placed at 4 °C for 3–4 h. The content of the tube was precipitated with ammonium sulphate (till 50% saturation), and the sedimented VLPs were dissolved in working buffer (20 mM Tris-HCl, 5 mM EDTA, 0.65 M NaCl, pH 7.8) and fractionated on a Sepharose CL-4B (Sigma Aldrich) column. Specifically, yeast-produced wt HBc and HBc-K80 VLPs were placed onto a larger column (1.4 × 70 cm) at a velocity 2 ml/h (90 min/3 ml fraction), and the bacteria-produced wt HBc and HBc-K75 VLPs were placed onto a smaller column (1 × 40 cm) at a velocity 2 ml/h (60 min/2 ml fraction).

### Detection of protein and nucleic acids

All optical density measurements were performed using WPA BioWave S2100 (Biochrom Ltd., UK), SmartSpec™ Plus (BioRad) and Nanodrop ND-1000 (Thermo Scientific, USA) spectrophotometers. The amount of protein (in the presence of nucleic acids) was estimated according to^73^. To calculate that one optical absorbance unit of empty VLPs corresponds to 0.71 mg of HBc protein, the Vector NTI 10.0.1 (Invitrogen) software package was used.

### VLP analysis

The VLP preparations were analysed with respect to the presence of protein and nucleic acids using native agarose gel electrophoresis (NAGE). For NAGE, 0.7% TopVision LE GQ Agarose (Fermentas) in TAE buffer (40 mM Tris, 20 mM acetic acid, 1 mM EDTA) supplemented with 1 μg/ml ethidium bromide was used with subsequent Coomassie Blue R-250 (60 μg/ml of Coomassie Blue R-250 in 10% acetic acid) staining of the gels. The following buffers were used to create the different pH conditions: 0.1 M sodium acetate/acetic acid at pH 5.3; 0.1 M K_2_PO_4_/NaOH at pH 6.5 and pH 7.5; TAE buffer at pH 8.3; and 0.025 M Na_2_B_4_O_7_ × 10H_2_O/HCl at pH 9.0 (according^74^).

Protein samples were analysed on 15% SDS-PAGE gels in a Tris-glycine-SDS system with subsequent Coomassie or silver staining according to standard procedures (LKB Laboratory Manual).

RNA analysis was performed using formaldehyde agarose gel electrophoresis (FAGE) and also on a 2100 Bioanalyser (Agilent Technologies) with the RNA Analysis Kit. To determine the RNA size and to enable comparisons between the different RNA samples, internal markers were used.

### Electron microscopy and dynamic light scattering analysis

For electron microscopy, VLPs in suspension were adsorbed to carbon-formvar coated copper grids and negatively stained with a 1% uranyl acetate aqueous solution. The grids were examined with a JEM-1230 electron microscope (Jeol Ltd., Tokyo, Japan) at 100 kV.

The size of the particles was detected by dynamic light scattering (DLS) in a Zetasizer Nano ZS (Malvern Instruments Ltd, UK) instrument. Histograms based on the intensity and volume parameters were drawn and statistics were calculated from data files using the software provided with apparatus.

### VLP packaging

Contact packaging was conducted by mixing empty HBc particles with nucleic acid material in working buffer (PBS is also suitable) and placing the mixture at ambient temperature for 10–15 min. The reconstruction reaction for plasmids and long DNA fragment packaging in empty HBc VLPs was conducted by dialysing the desired mixture against 7 M urea + 1 M NaCl at 4 °C for one hour. Next, dialysis against the restoration buffer was performed for one hour and then a fresh portion of restoration buffer was added and dialysed overnight.

### CsCl density gradient

The empty core and DNA plasmid or DNA fragment complexes were purified on a preformed CsCl gradient. The gradient consisted of two 6 ml layers in a 12 ml polyallomer tube. The bottom layer was taken from ready solution (44 g CsCl + 60 ml working buffer), and the upper layer consisted of protein to purify, 2.2 g of CsCl and working buffer. After centrifugation in a Beckman Coulter Optima L-100XP ultracentrifuge (rotor SW32 Ti) at 20 500 rpm for 13 h at 4 °C, the tube was pierced, and 0.5 ml fractions were collected. After OD measurements and fraction NAGE, the fractions were dialysed, concentrated in a dialysis tube using dry Sephadex G-100 (GE Healthcare Life Sciences) or directly used for further experiments.

### Sucrose density gradient

RNA or DNA complexes with VLPs were loaded onto a pre-formed 5–36% sucrose gradient in Polyallomer 14 × 95 mm (12 ml) tubes (following sucrose concentrations (w/v) of the layers: 36% - 1 ml; 30% - 1 ml; 25% - 2 ml; 20% - 2.7 ml; 15% - 2 ml; 10% - 2 ml; 5% - 1 ml) for centrifugation in a Beckman Coulter Optima L-100XP ultracentrifuge (rotor SW32 Ti) at 20,500 rpm for 13 h at 4 °C. Next, 0.5-ml fractions were collected from the pierced bottom of the tube.

## Additional Information

**How to cite this article**: Strods, A. *et al.* Preparation by alkaline treatment and detailed characterisation of empty hepatitis B virus core particles for vaccine and gene therapy applications. *Sci. Rep.* **5**, 11639; doi: 10.1038/srep11639 (2015).

## Supplementary Material

Supplementary Information

Supplementary Information
