# Supplementary material for: Preparation by alkaline treatment and detailed characterisation of empty hepatitis B virus core particles for vaccine and gene therapy applications
Source: Sci Rep. 2015 Jun 26;5:11639. doi: 10.1038/srep11639 (PMC4650659; doi:10.1038/srep11639)
Supplement: Supplementary Information [file srep11639-s1.pdf]

SUPPLEMENTARY INFORMATION

Preparation by alkaline treatment and detailed characterisation of empty hepatitis B virus core particles for vaccine and gene therapy applications

Arnis Strods, Velta Ose, Janis Bogans, Indulis Cielens, Gints Kalnins, Ilze Radovica, Andris Kazaks, Paul Pumpens, Regina Renhofa*

Preparation of HBc VLPs by traditional column chromatography methods before alkaline treatment

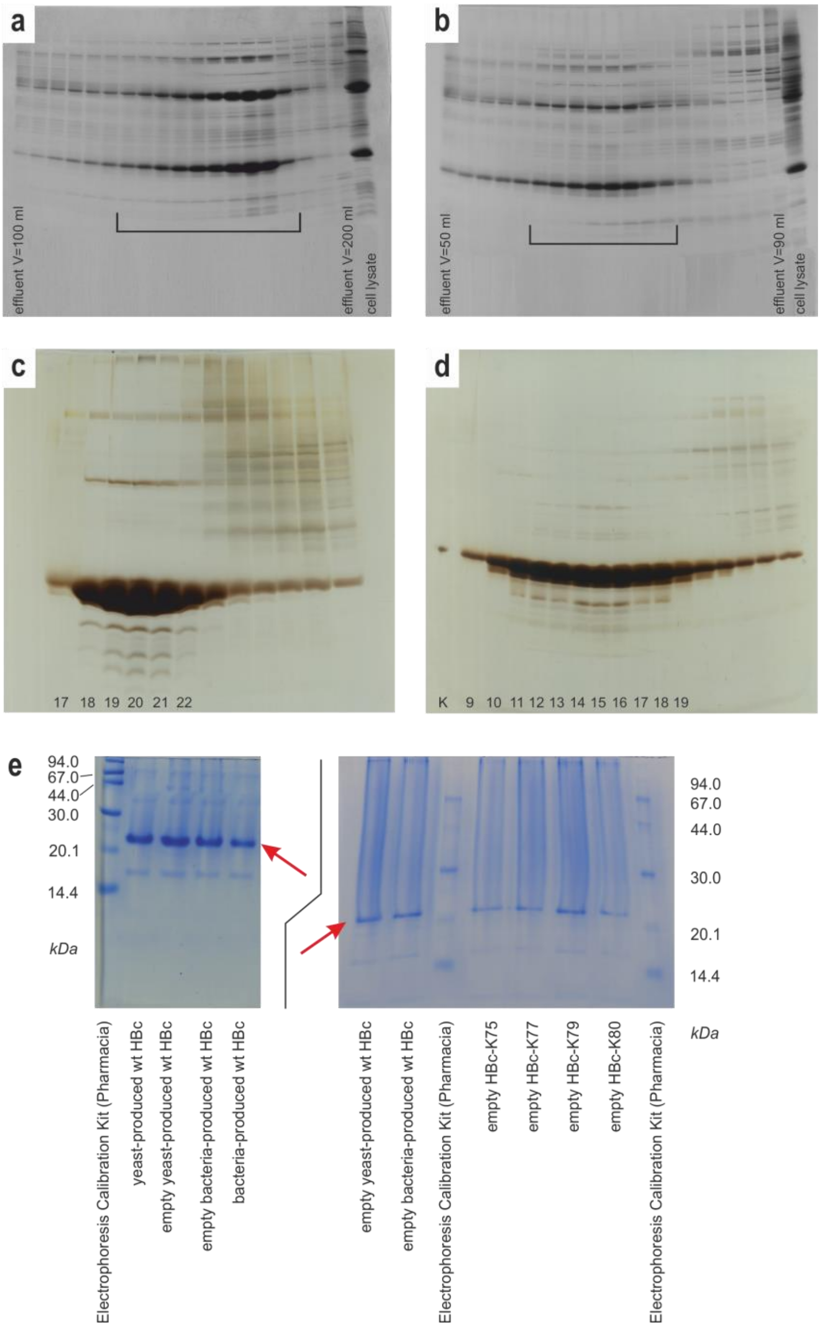

Fig. S1. Preparation of HBc VLPs by traditional column chromatography methods before alkaline treatment – silver-stained PAGE with typical profiles of successive column chromatography steps during the purification of bacteria-produced wt HBc VLPs before alkaline treatment. (a) Sepharose CL-2B, (b) Sepharose CL4B, (c) first Sephacryl S300, (d) second Sephacryl S300. Fractions collected fur further purification steps are designated by brackets (a,b) or by fraction numbers (c,d). K – control. (e) Coomassie-stained PAGE with purified wt HBc VLPs before alkaline treatment and wt HBc VLPs and lysine-exposing HBc VLPs after alkaline treatment. Red arrow points to the HBV core protein.

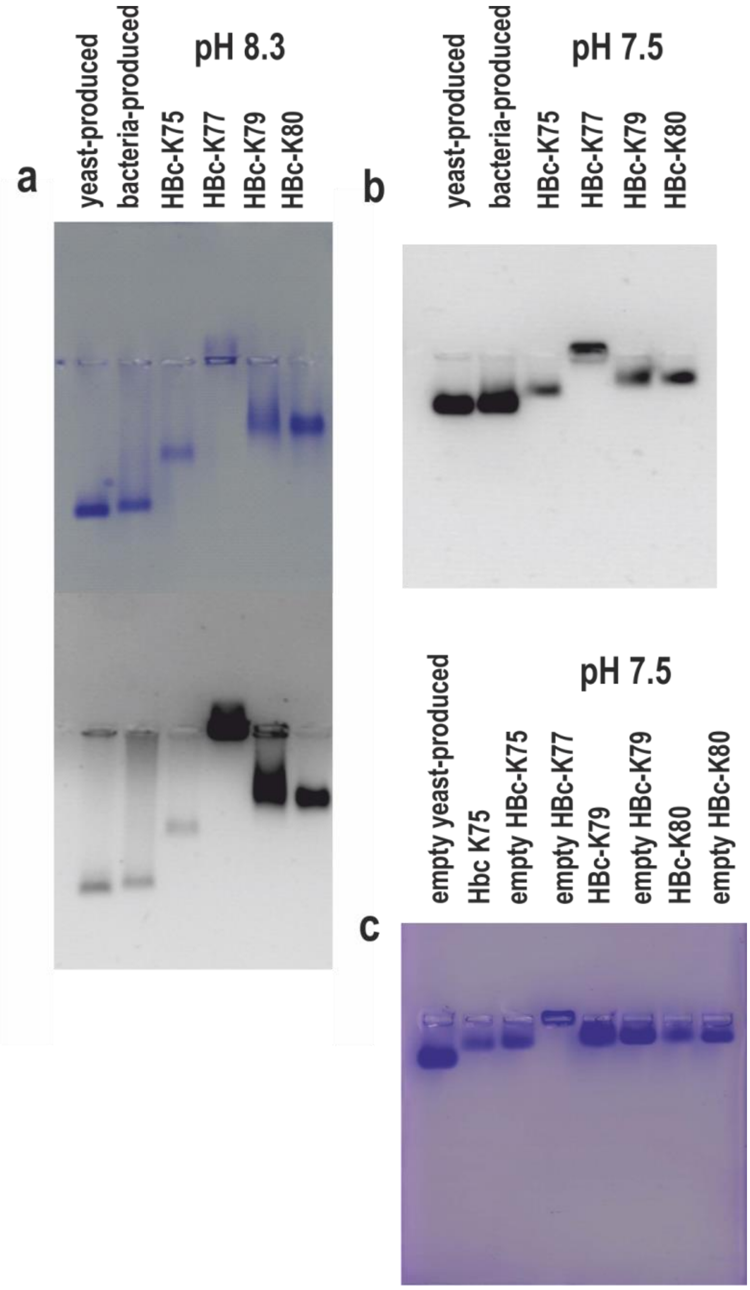

Fig. S2. HBc VLP variants purified before and after alkaline treatment. (a) NAGE analysis at pH 8.3 by Coomassie (top) and ethidium bromide (bottom) staining; (b) NAGE at pH 7.5 by ethidium bromide staining; (c) NAGE at pH 7.5 by Coomassie staining.

RNA content of purified HBc VLP variants before alkali treatment

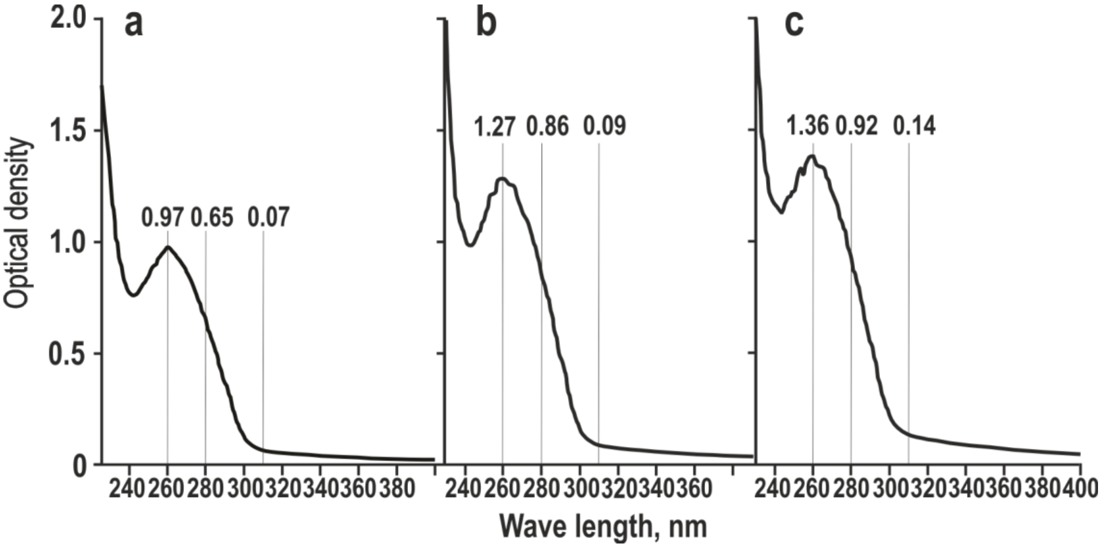

Fig. S3. Typical UV spectra of HBc VLP variants before alkaline treatment. (a) Bacteria-produced wt HBc VLPs, (b) yeast-produced wt HBc VLPs, (c) HBc-K79. The OD values at the wave lengths of 260, 280, and 310 nm are indicated.

Determination of length profile of RNA fragments encapsidated by HBc VLPs

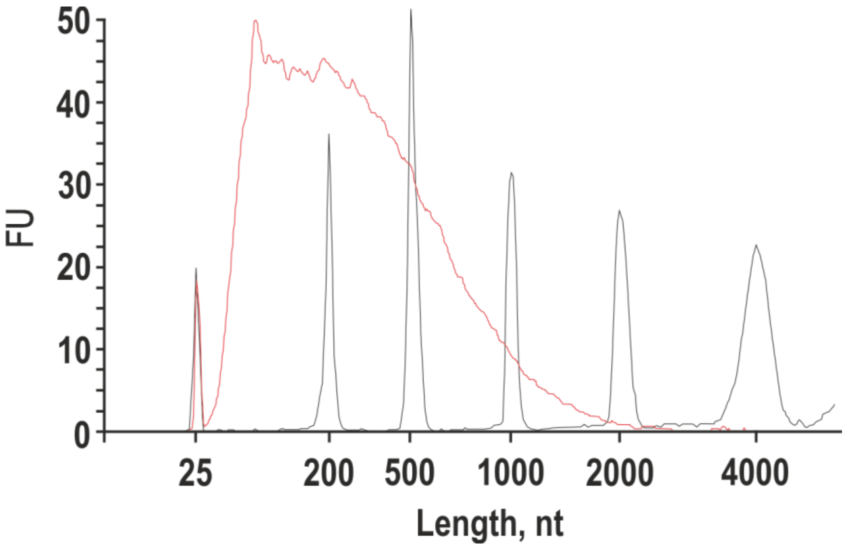

Fig. S4. Capillary electrophoresis of RNA fragments encapsidated by bacteria-produced wt HBc VLPs. Length profile of encapsidated RNA (red) and control polynucleotide ladder (black).

Detection of RNA encapsidated by HBc VLPs

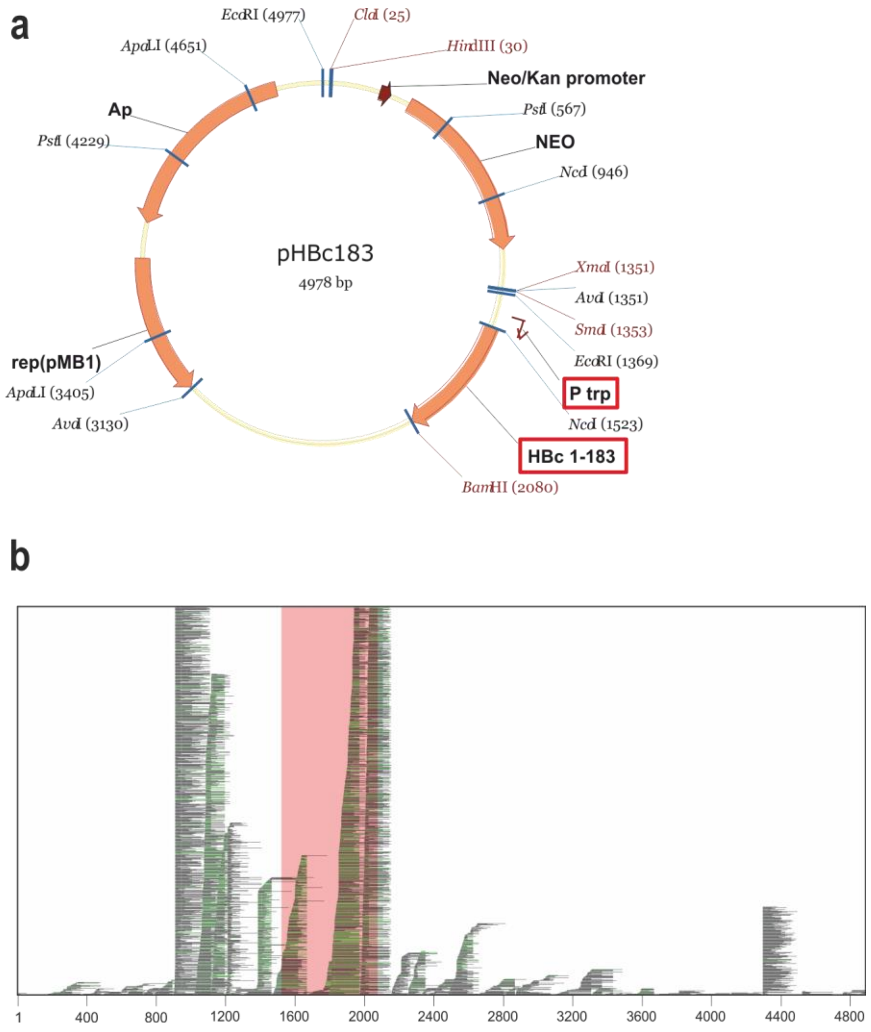

Fig. S5. Sequencing of RNA encapsidated by bacteria-produced wt HBc VLPs. (a) Map of the pHBc183 plasmid encoding HBc gene at positions 1524 to 2072, total 549 bp, or 183aa, (b) alignment of PGM sequencing data to the pHBc183 plasmid. The HBc gene-encoding region is marked red.

Characterisation of empty HBc particles

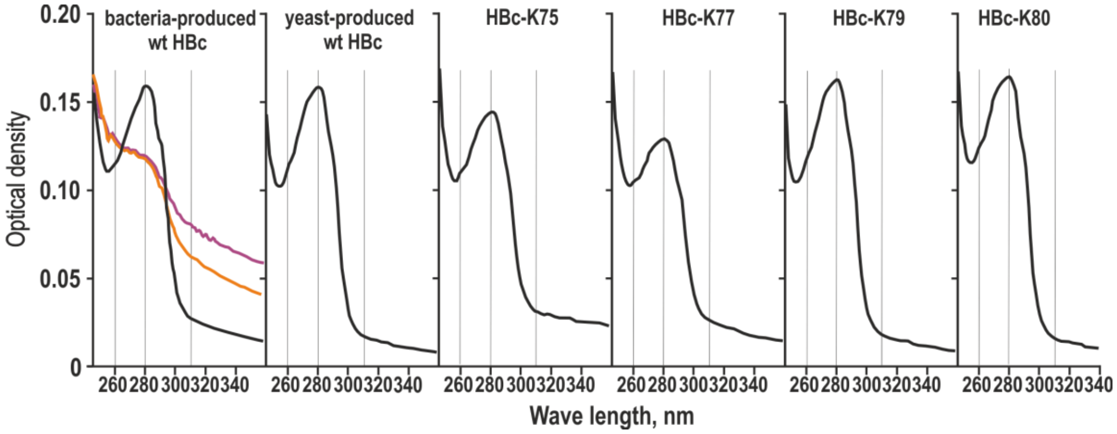

Fig. S6. Typical UV spectra of fractions B containing empty wt HBc VLPs from *E. coli* and *P. pastoris*, as well as of VLPs formed by four lysine-exposing HBc variants. UV spectra of fraction A of the bacteria-produced wt HBc VLPs in water (red) or urea (orange) are added for reference to the respective plot (left). OD positions at the wavelengths of 260, 280, and 310 nm are indicated by vertical lines.

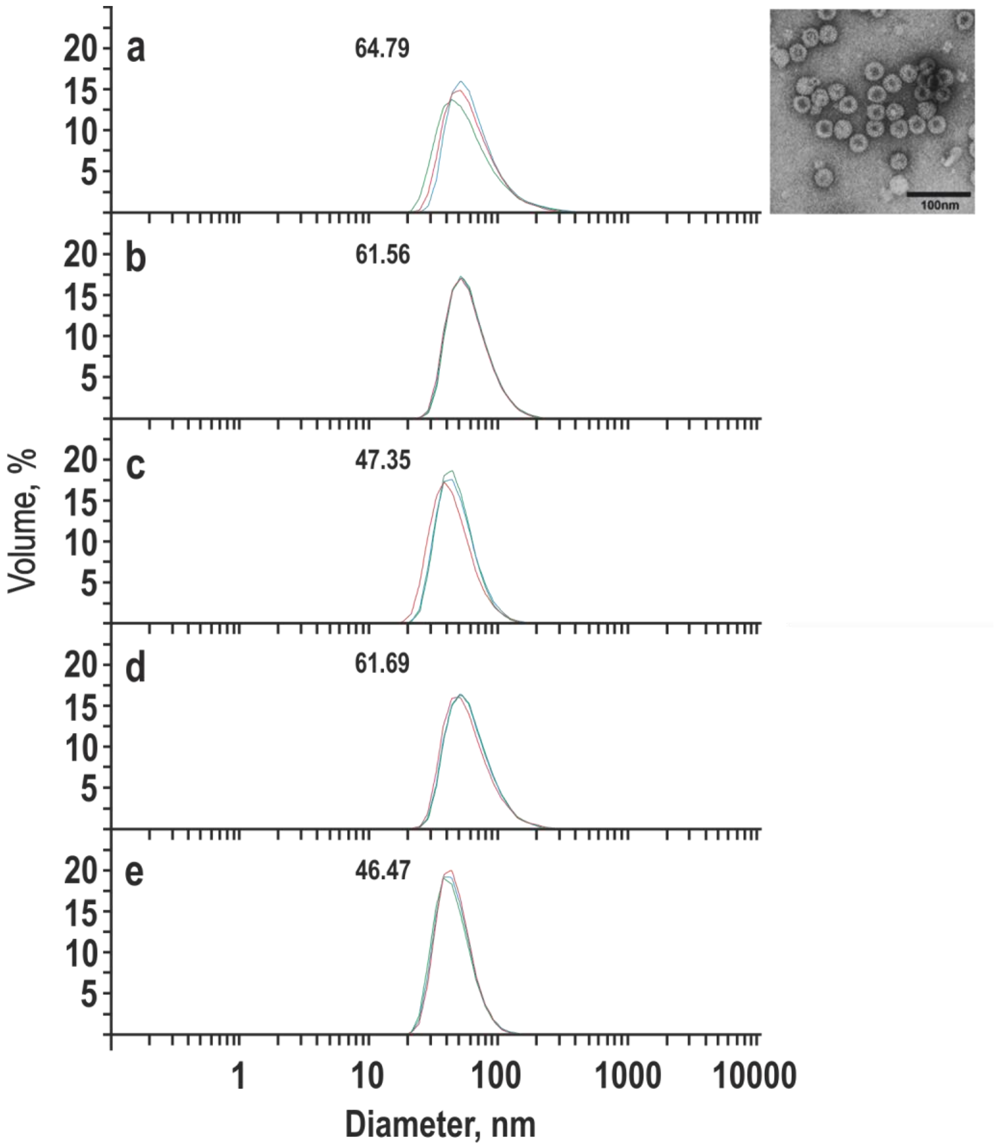

Fig. S7. Characterisation of the intermediate fraction pools of the alkali-treated HBc VLP variants by DLS (left) and EM (right). (a) HBc-K77, fraction C, (b) HBc-K79, fraction C, (c) HBc-K79, fraction D, (d) HBc-K80, fraction C, and (e) HBc-K80, fraction D.

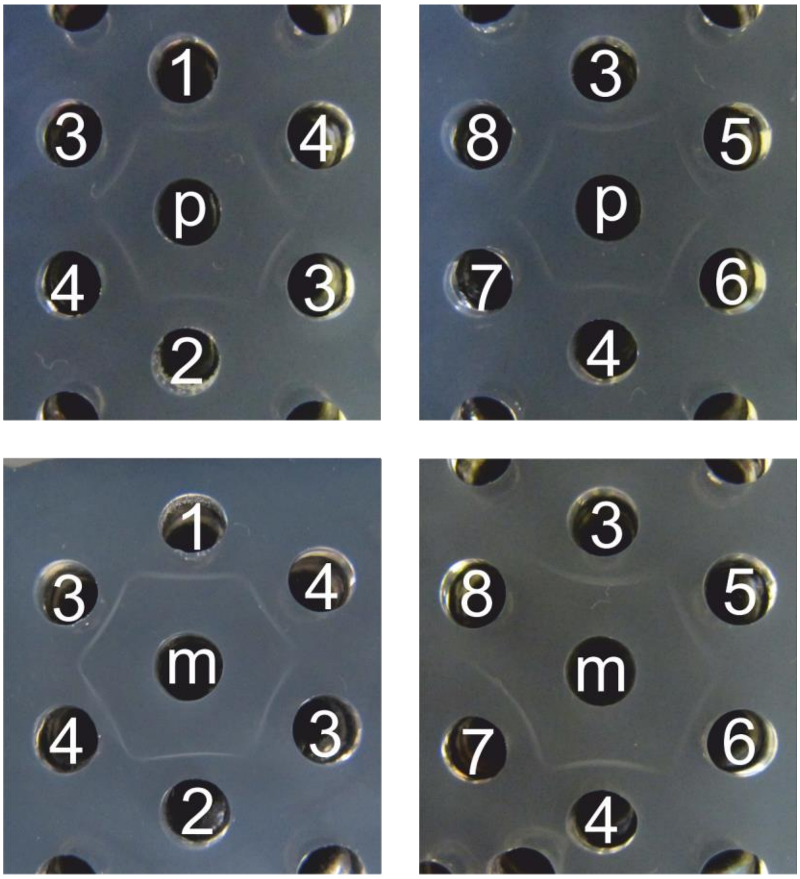

Fig. S8. Ouchterlony's double radial immune diffusion test with polyclonal rabbit antibodies (p) and monoclonal C1-5 antibodies recognizing 78-DPIxxD-83 epitope (m) and HBc VLPs: bacteria-produced wt HBc (1), yeast-produced HBc (2), empty bacteria-produced wt HBc (3), empty yeast-produced HBc (4), empty HBc-K75 (5), empty HBc-K77 (6), empty HBc-K79 (7), empty HBc-K80 (8). Diffusion was performed by undiluted antibodies and VLPs at 1.5 mg/ml concentrations for 48 hours in 0.5% agarose gel (in working buffer) placed in a humidity box.

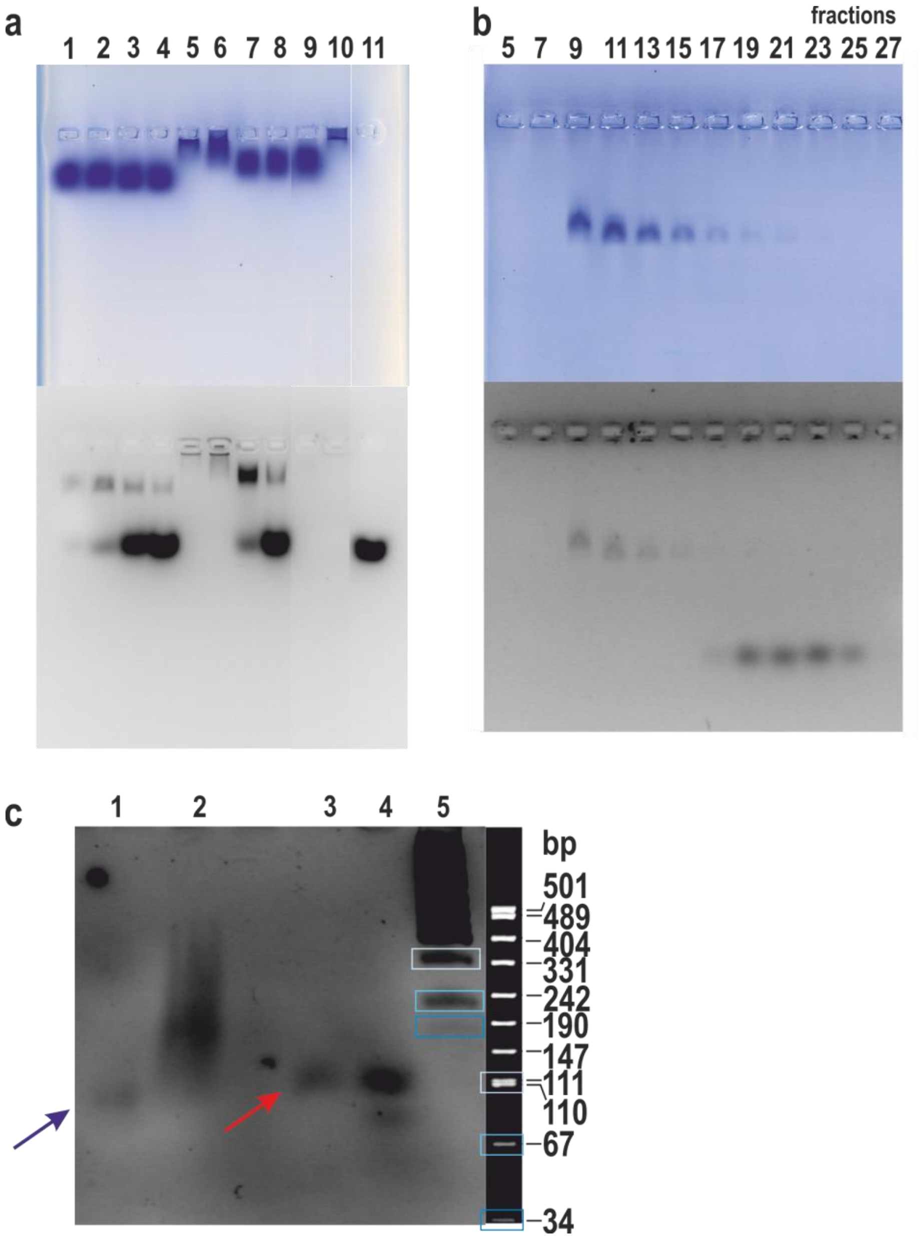

Fig. S9. Encapsidation of tRNA by contact of empty bacteria- and yeast-produced wt HBc VLPs. (a) Titration of yeast-produced (1-4) and bacteria-produced (5-8) wt HBc VLPs by tRNA at different VLP/tRNA molar ratios: 1:2.5 (1 and 5), 1:10 (2 and 6), 1:25 (3 and 7), and 1:50 (4 and 8); empty yeast-produced (9) and bacteria-produced (10) wt HBc VLPs, as well as tRNA before packaging (11) are loaded on the gel as controls. (b) Gel filtration of tRNA-packaged yeast-produced wt HBc VLPs on a Ultrogel A2 (LKB) column, elution with working buffer (0.5 ml/fraction). Appropriate fractions are shown in NAGE after coomassie (top) and ethidium bromide (bottom) staining. (c) PAGE of phenol-extracted tRNA from the pooled 8-14 fractions (see B) (1) and non-encapsidated control tRNA (2), (3) and (4) are empty yeast-produced HBc VLPs packed with ODN and ODN alone, respectively, (5) pUC19/MspI DNA ladder as a marker. Blue arrow denotes degraded tRNA and red arrow points to ODN.

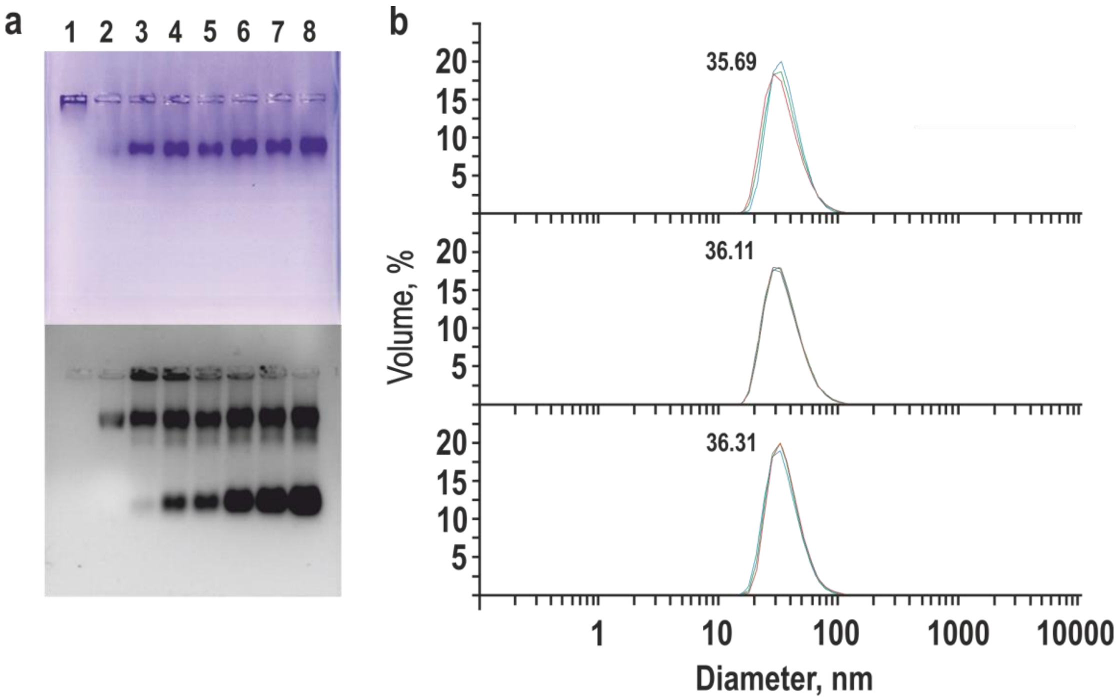

Fig. S10. Encapsidation of rRNA by contact of empty HBc-K75 VLPs. (a) Titration of HBc-K75 VLPs by rRNA at different mononucleotide amount per one VLP: 1600 nt (3), 3200 nt (4), 4800 nt (5), 6400 nt (6), 8000 nt (7), 9600 nt (8); empty HBc-K75 (1) and initial alkaline non-treated HBc-K75 (2) VLPs were used as controls. Coomassie (top) and ethidium bromide (bottom) staining of NAGE patterns. (b) DLS analysis of rRNA-packaged HBc-K75 VLPs (top) by comparison with empty (middle) and initial bacteria-produced (bottom) HBc-K75 VLPs.

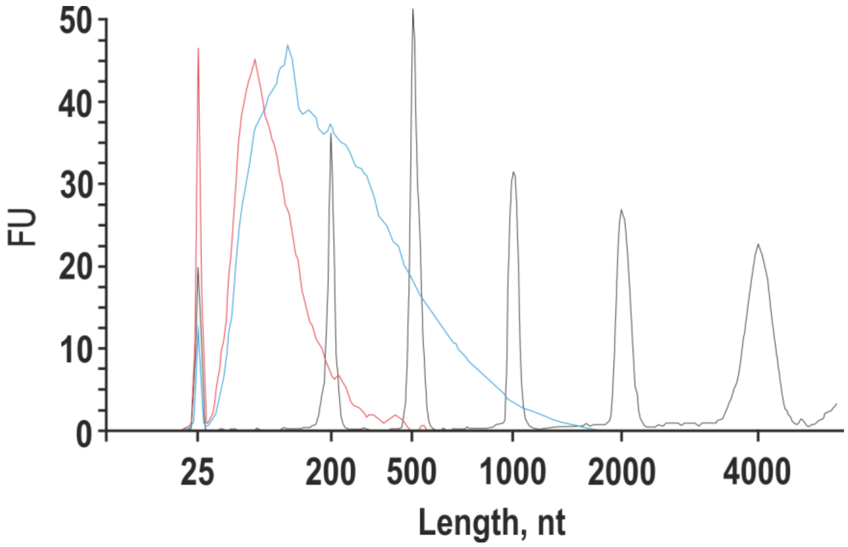

Fig. S11. Fate of rRNA encapsidated by empty HBc-K79 VLPs. Electrophoretic separation with capillary electrophoresis on a 2100 Bioanalyser of rRNA before packaging (blue) and unpacked rRNA (red). Molecular mass of the packaged RNAs is determined by comparison with the ladder (black).

Preparation and characterization of DTA mRNA

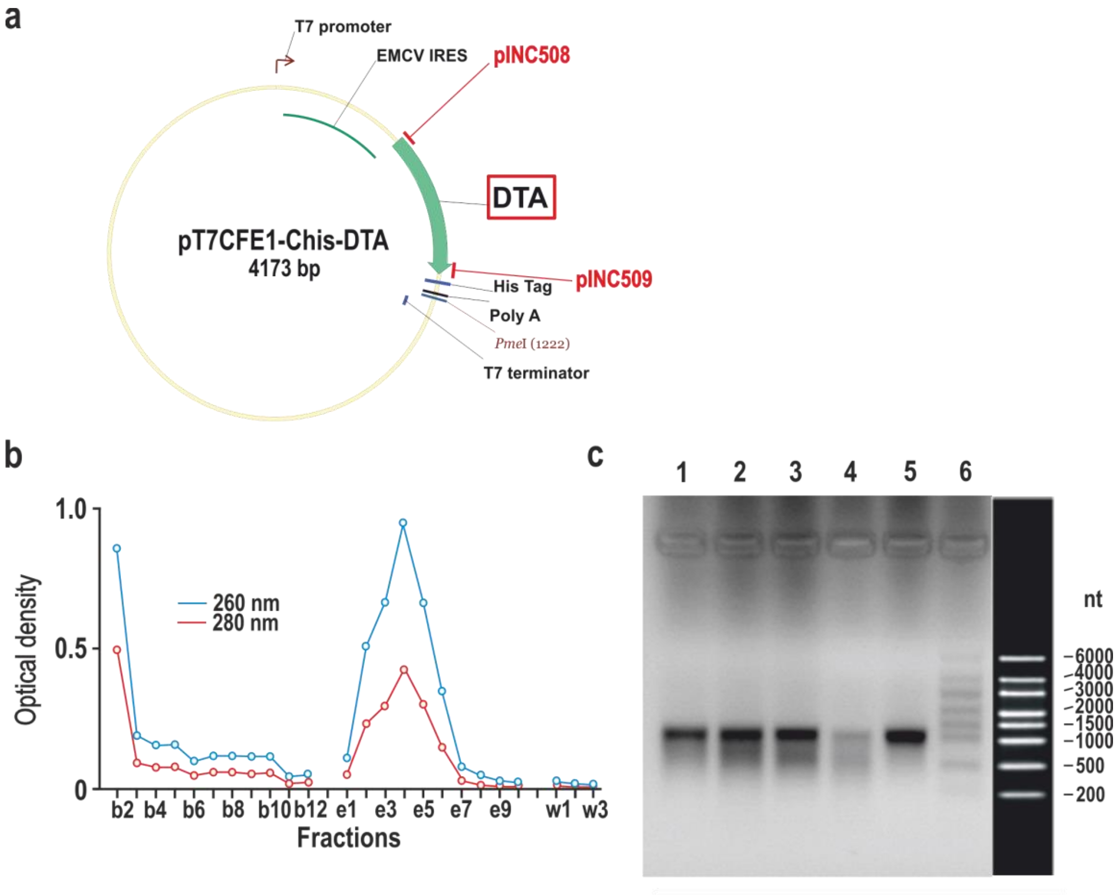

Fig. S12. Preparation and characterisation of DTA mRNA. (a) Map of the plasmid encoding DTA gene at positions 528 to 1132, total 585 bp or 195 aa. (b) Purification of DTA mRNA on a oligo(dT) cellulose column. (c) FAGE monitoring of the purification and characterisation of the DTA mRNA: oligo(dT) cellulose pooled binding fractions b1-b3 (1) and b4-b11 (2), pooled elution fractions e1-e9 (further used for packaging) (3), pooled water washes w1-w3 (4), transcribed mRNA (5), and RiboRuler™ RNA Ladder, High Range (6,7) as the marker.

Encapsidation of mRNA by direct contact with empty HBc VLPs

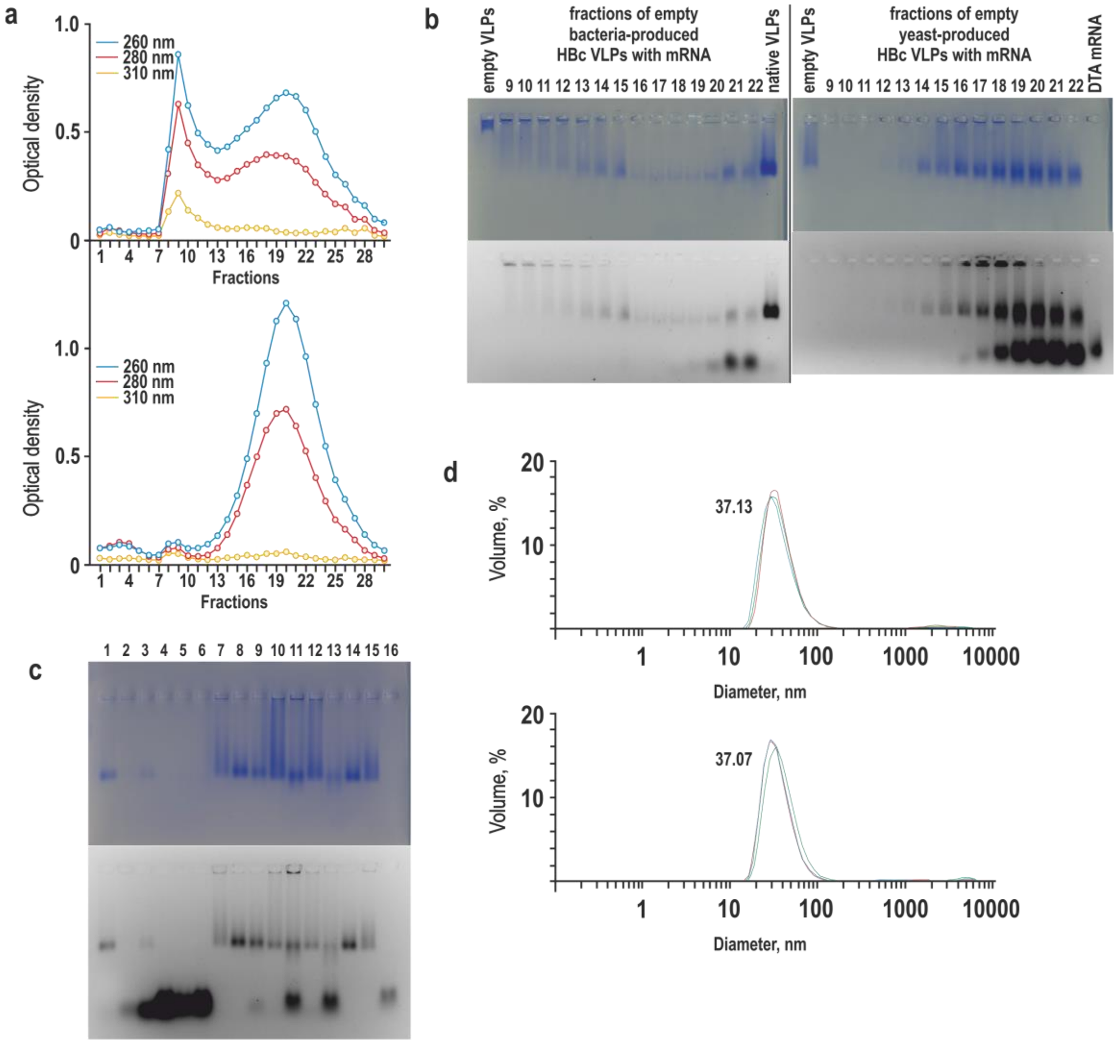

Fig. S13. Contact packaging of empty wt HBc VLPs by DTA mRNA at a molar ratio 1:2. (a) Sepharose CL-2B column chromatography of bacteria- (top) and yeast- (bottom) produced wt HBc VLPs, (b) NAGE of Sepharose CL-2B column fractions after the separation of bacteria- (left) and yeast- (right) produced wt HBc VLPs stained by Coomassie blue (top) and ethidium bromide (bottom), initial alkaline non-treated HBc bacteria-produced wt HBc VLPs, and alkali-treated HBc VLPs (bacteria- or yeast-produced, respectively) are used as a control. (c) NAGE of Sepharose CL-2B column chromatography fractions after ammonium sulphate precipitation: bacteria-produced HBc VLP fractions 8-14 (2, 7), 15, 18-20 (3, 8), 21-24 (4, 9, 12), yeast-produced HBc VLP fractions 16-17 (5, 10), 18-24 (6, 11, 13) after rinsing with water, working buffer and additional portion of working buffer, respectively; bacteria-produced HBc VLP fractions 21-24 (14) and yeast-produced HBc VLP fractions 18-24 (15) after multiple rinses with water; bacteria-produced wt HBc VLPs (1) and DTA mRNA (16) are used as a control. (d) DLS analysis of contact packaged empty wt HBc VLPs with DTA mRNA after Sepharose CL-2B chromatography and precipitation with ammonium sulphate to remove free mRNA: bacteria-produced, rinsed fractions with A260/A280 ratio 1.43 (top) and yeast-produced, rinsed fractions with A260/A280 ratio 1.15 (bottom).

Fine analysis of DTA mRNA isolated from HBc VLPs: sequencing of DTA mRNA isolated from HBc-K80 VLPs

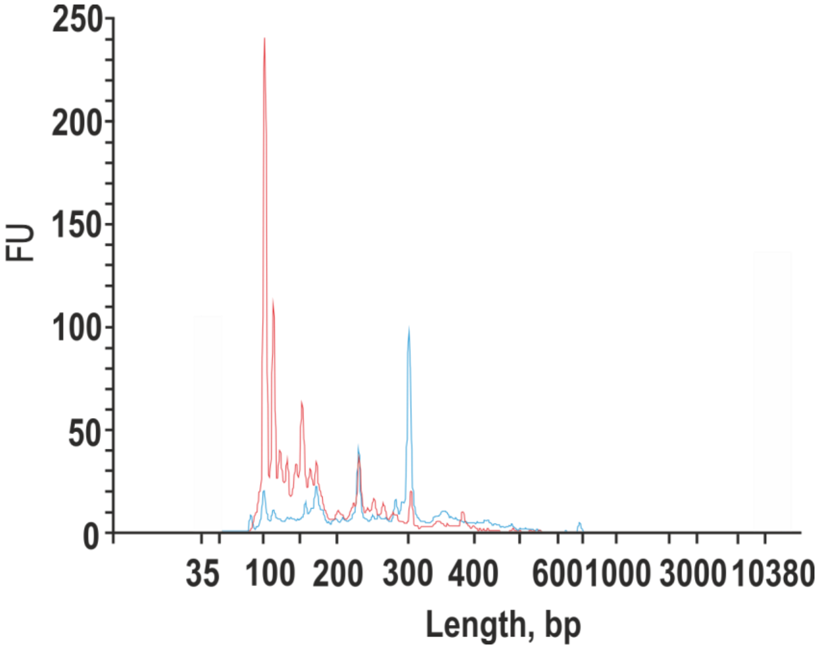

Fig. S14. Length of cDNA libraries prepared for the DTA mRNA sequencing by the BioAnalyzer 2100. Libraries of DTA mRNA before encapsidation (blue) and recovered from the mRNA-packaged HBc-K80 VLPs (red).

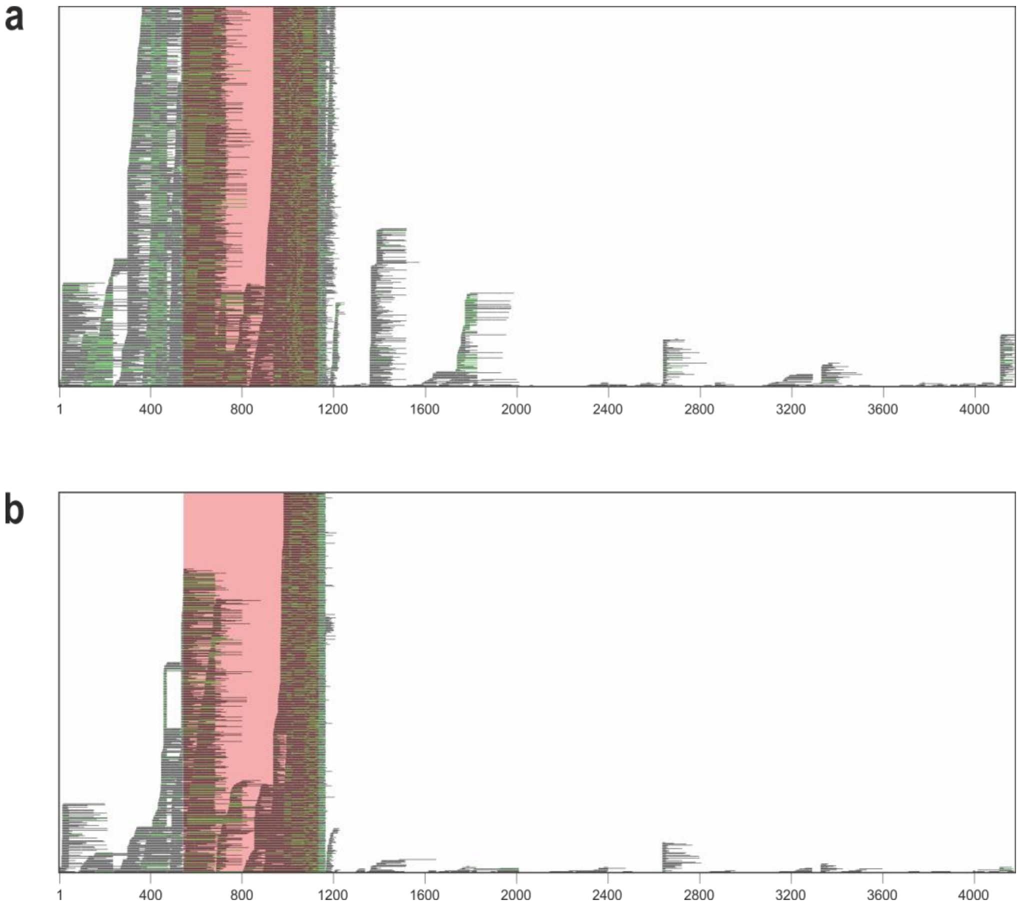

Fig. S15. Alignment of the nucleic acid material extracted from the DTA mRNA-packaged HBc-K80 VLPs and sequenced via the PGM technique and alignment to the pT7CFE1-Chis-DTA plasmid encoding DTA mRNA. (A) Non-packaged DTA mRNA as a control, (B) HBc-K80 VLP-packaged DTA mRNA. The DTA mRNA-encoding sequence is marked red.

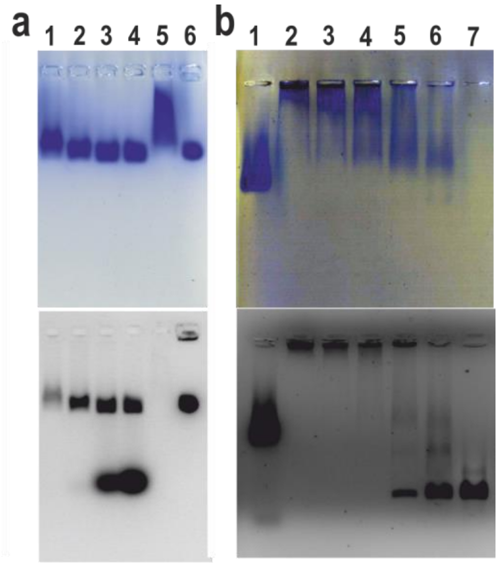

Fig. S16. Contact packaging of yeast-produced wt HBc VLPs by 20 nt CpG ODNs and bacteria-produced wt HBc VLPs by a 601 bp DNA fragment. (a) Titration of yeast-produced wt HBc VLPs by ODN20 at appropriate VLP molar superiority over ODN: 15 (1), 31 (2), 76 (3), 153 (4), empty (5) and non-alkali treated (6) yeast-produced wt HBc VLPs as controls, (b) titration of a 601 bp DNA fragment by empty bacteria-produced wt HBc VLPs in the corresponding molar ratios: 1:15 (2), 1:10 (3), 1:5 (4), 1:2 (5), 1:1 (6); 601 bp DNA fragment (7) and bacteria-produced wt HBc VLPs (1) as a controls.

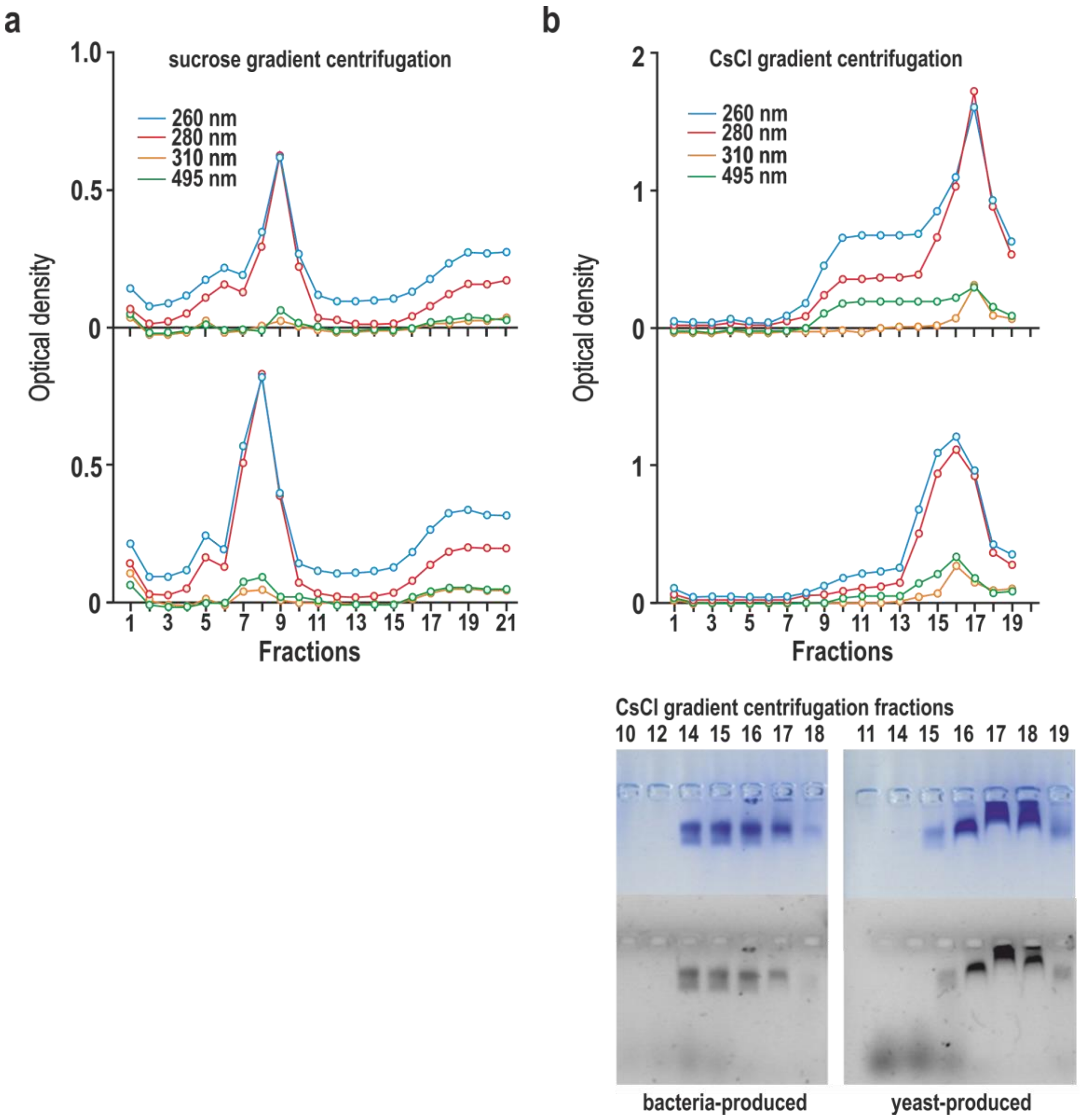

Fig. S17. Stability of bacteria- and yeast-produced wt HBc VLPs carrying encapsidated ODN20. (a) Sucrose gradient centrifugation profiles of bacteria- (top) and yeast- (bottom) produced wt HBc VLPs carrying encapsidated ODN20, (b) CsCl density gradient centrifugation of bacteria- (top) and yeast- (bottom) produced HBc VLPs carrying encapsidated ODN20 and NAGE of the respective gradient fractions stained by Coomassie (top) and ethidium bromide (bottom).

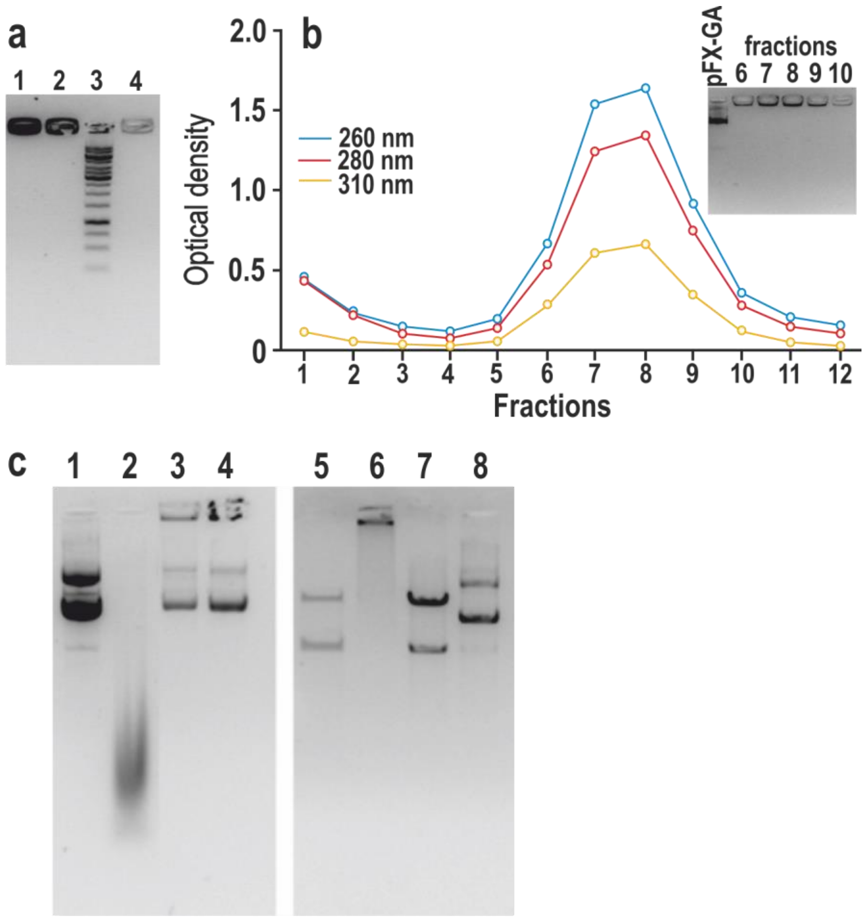

Fig. S18. Restoration of wt HBc VLPs after 7 M urea treatment by full-length plasmid DNA. Yeast-produced wt HBc VLPs restored by pFX-GA plasmid in 34-fold molar ratio of VLP versus plasmid: (a) NAGE of restored VLPs without (1) or with (2) Mg^{2+} and 1 kb ladder (3) and empty VLPs (4) as controls; (b) Sepharose CL-2B column chromatography profile for the Mg^{2+}-deficient restoration variant and NAGE of the respective column fractions in the insert. (c) Phenol extraction of the encapsidated DNA from bacteria-produced (1-4) or yeast-produced (5-8) HBc VLPs: extracted pCEP-CXCR4-eGFP plasmid without (3) and with (4) DNase treatment before extraction; extracted SalI fragments of pCEP-CXCR4-eGFP plasmid (5), controls: pCEP-CXCR4-eGFP plasmid (1,8), DNase treated pCEP-CXCR4-eGFP plasmid (2), empty yeast-produced HBc VLPs packaged with SalI fragments of pCEP-CXCR4-eGFP plasmid (6), SalI fragments of pCEP-CXCR4-eGFP plasmid (7). Reconstruction ratios of VLPs versus pCEP-CXCR4-eGFP plasmid and SalI fragments of pCEP-CXCR4-eGFP plasmid were 1:15 and 1:20, respectively.

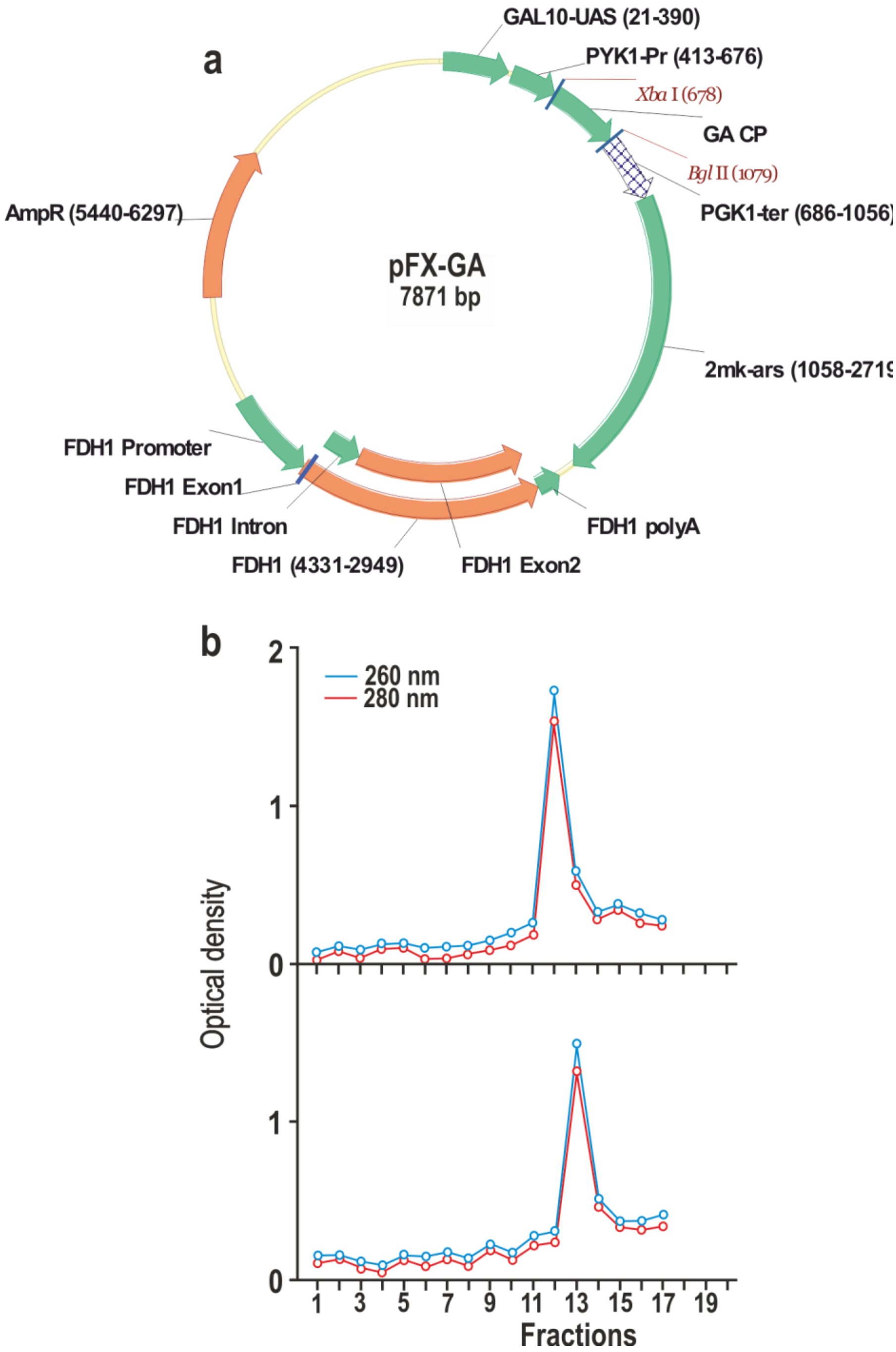

Fig. S19. Restoration (after 7 M urea treatment) of yeast-produced wt HBc VLPs in 34-fold superiority over pFX-GA plasmid DNA. (a) Map of pFX-GA plasmid. (b) CsCl density gradient centrifugation of the restored VLPs in the absence (top) and in the presence (bottom) of 5 mM Mg^{2+}.

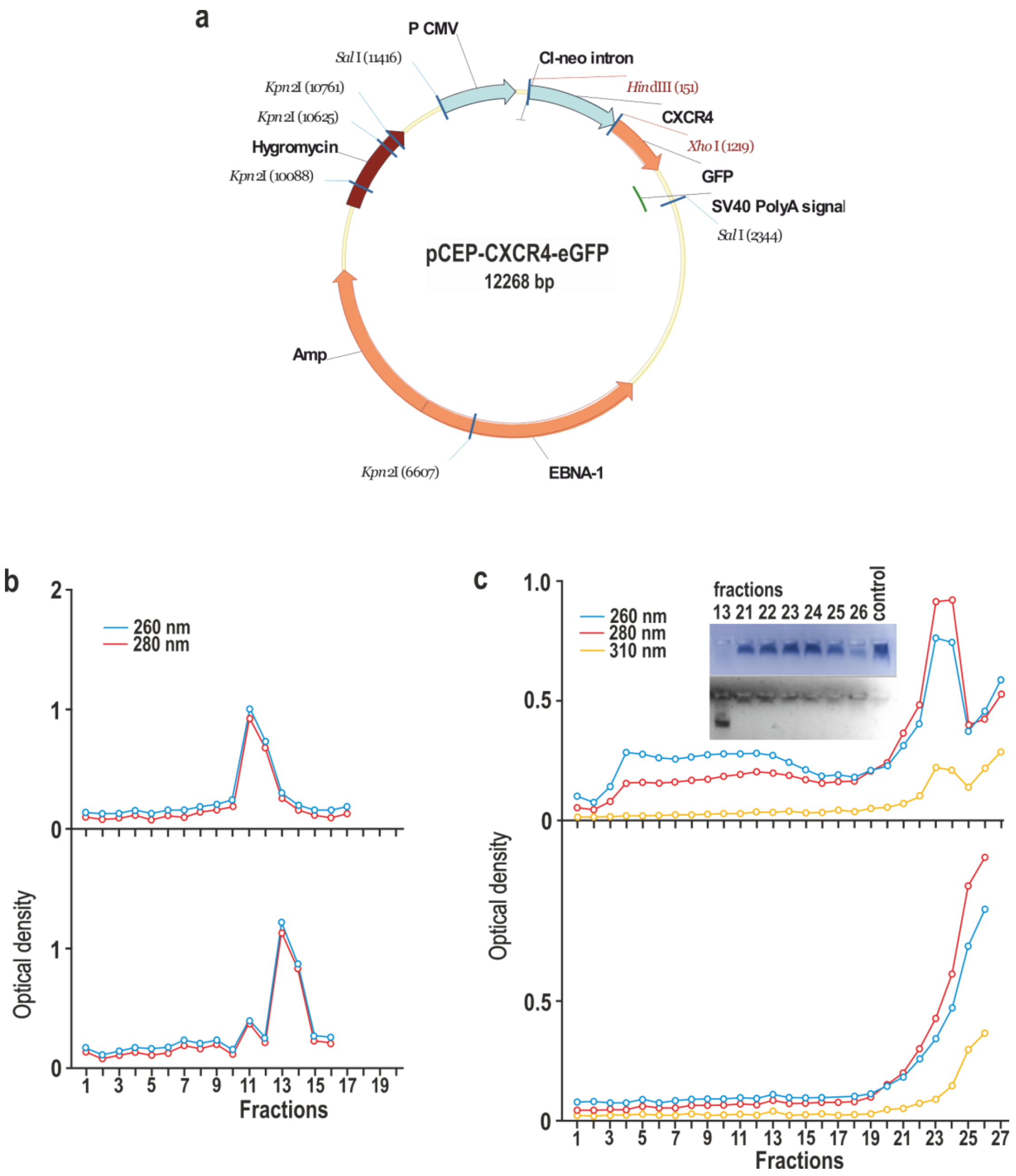

Fig. S20. Restoration of yeast-produced wt HBc and HBc-K79 VLPs after 7 M urea treatment by pCEP-CXCR4-eGFP plasmid DNA. (A) Map of pCEP-CXCR4-eGFP plasmid. (B) CsCl density gradient centrifugation of the restored yeast-produced wt HBc VLPs with pCEP-CXCR4-eGFP plasmid linearised by HindIII (top) and split into two fragments by SalI (bottom) in 53-fold molar ratio of VLP: plasmid. (C) CsCl density gradient centrifugation of the restored HBc-K79 by mixture of pCEP-CXCR4-eGFP fragments of 3481 and 4263 bp in length in 5-fold molar ratio of VLP: plasmid (top) and empty HBc-K79 VLPs as a control (bottom). NAGE analysis of the respective gradient fractions stained by Coomassie (top) and Ethidium bromide (bottom) is shown in the insert.

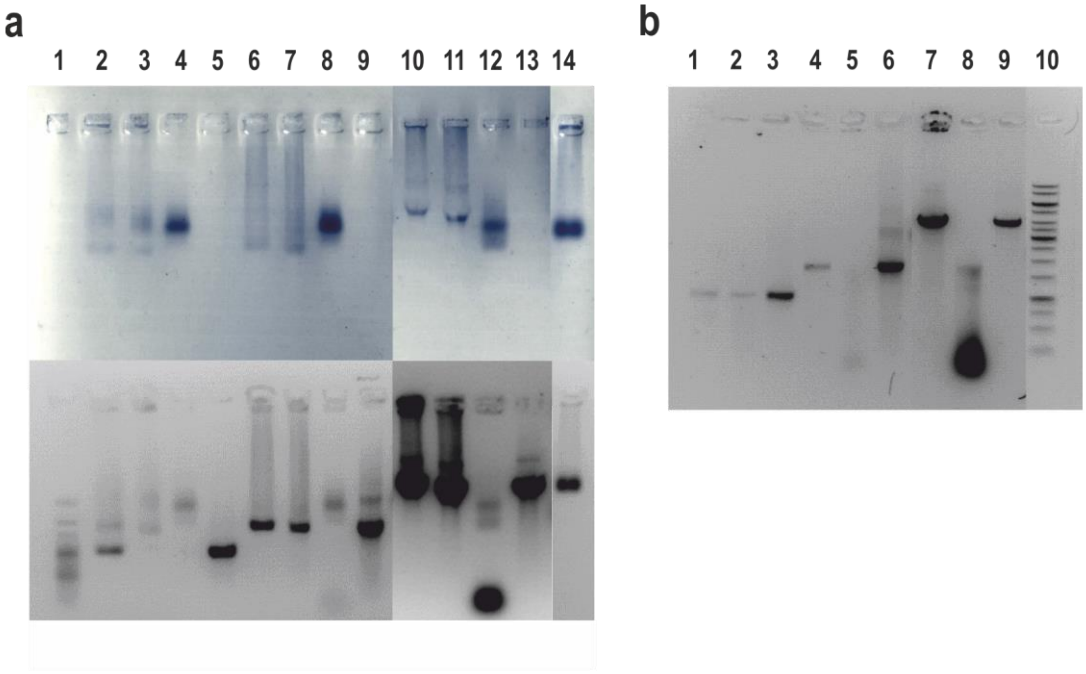

Fig. S21. NAGE analysis of the restoration of bacteria-produced wt HBc VLPs by DNA fragments 1047, 1811, and 4182 bp in length. (a) Coomassie- (top) and ethidium bromide- (bottom) stained gels of the restored encapsidated HBc VLPs (all purified by CsCl density gradient centrifugation) at the fragment to HBc VLP molar ratio 1:1.5 in the case of the fragments 1047 bp (2-5), 1811 bp (6-9), and linearised pT7CFE-Chis-DTA plasmid of 4182 bp (10-12) where samples (2,6,10) are in a restoration buffer, (3,7,11) are in DNase buffer, and (4,8,12) are samples in DNAse buffer and treated by DNase; controls: 100 bp Plus DNA ladder (1), the respective DNA fragments (5,9,13), and initial alkali non-treated bacteria-produced wt HBc VLPs (14). (b) Ethidium bromide-stained FAGE of phenol-extracted content of the restored HBc VLPs carrying fragments 1047 bp (1-3), 1811 bp (4-6), and linearised pT7CFE-Chis-DTA plasmid of 4182 bp (7-9) where the encapsidated VLPs were not treated (1,4,7) or treated with DNase (2,5,8) before phenol extraction; controls: the respective DNA fragments (3,6,9), 1 kb ladder (10).

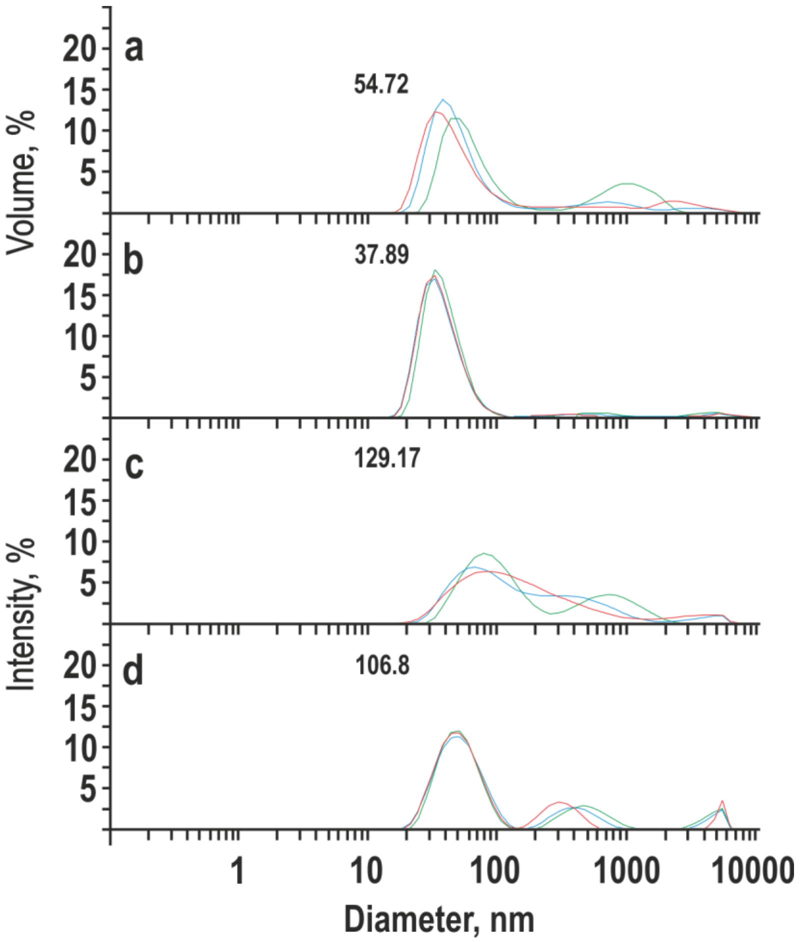

Fig. S22. DLS monitoring of the restoration of a 1047 bp DNA fragment by bacteria-produced wt HBc VLPs. DLS measurements using volume (top) or intensity (bottom) parameters were performed for the packaged VLPs before (a, c) and after (b, d) DNAse treatment.

Table S1. Summary of statistical NGS results, mapped against plasmid or *E.coli* genome sequences.

	**bacteria-produced HBc VLPs extracted content**		**mRNA**		**extracted content from DTA mRNA packaged in HBc-K80 particles**	
	mapped nt count	**percentage from total**	mapped nt count	**percentage from total**	mapped nt count	**percentage from total**
referred against pHBc183 plasmid sequence	1138106	**34,93%**	25712	**0,83%**	13657	**1,64%**
referred against pT7CFE1-Chis-DTA plasmid sequence			3041350	**98,65%**	723834	**86,77%**
referred against E.coli genome sequence	2120428	**65,07%**	15847	**0,51%**	96692	**11,59%**

Encapsidation of deoxyribonucleic acid by empty HBc VLPs

Table S2. List of DNA sources: plasmids, fragments, and ODNs used for the DNA encapsidation experiments with empty HBc VLPs

DNA source, bp	obtaining method	plasmid	restriction enzymes or primers for the obtaining of fragment
**Full-length plasmids**			
12268 bp	-	pCEP-CXCR4-eGFP	plasmid from previous experiments[1]
7871 bp	-	pFX-GA	plasmid from previous experiments[2]
4182 bp	-	pT7CFE-Chis-DTA	constructed by inserting diphtheria toxin sequence[3] in pT7CFE1-Chis vector
**DNA fragments**			
12268 bp	restriction	pCEP-CXCR4-eGFP	HindIII (linearized plasmid)
3196 + 9072 bp	restriction	pCEP-CXCR4-eGFP	SalI (mixture of two fragments)
3481 + 4263 bp	restriction	pCEP-CXCR4-eGFP	Kpn2I, SalI, XhoI (extracted the mixture of two fragments from agarose gel)
4182 bp	restriction	pT7CFE-Chis-DTA	XhoI (linearized plasmid)
1811 bp	PCR	pCEP-CXCR4-eGFP	pARS13 (5’- GGG AAG CTT GCT AGC ATG GAG GGG ATC AGT ATA TAC ACT TCA GAT AAC TAC ACC GAG GAA ATG-3’)<br>pJAR19 (5’- GAG CGG CCG CAA GCT TAC TTG TAC AGC TCG TCC AT-3’)
1737 bp	PCR	pCEP-CXCR4-eGFP	pARS13<br>pINC448 (5’- GAC CAT GTG ATC AGC AGC CTC GTT GGG GTC-3’)
1289 bp	PCR	pCEP-CXCR4-eGFP	pARS13<br>pINC446 (5’- CAC TGC ACG CCG TAG GAG AAG GTG GTC ACG AG-3’)
1047 bp	restriction	pT7CFE-Chis-DTA	HindIII, MssI (PmeI)
601 bp	PCR	pT7CFE-Chis-DTA	pINC508 (5’- TAC ACA TAT GGG CGC TGA TGA TGT TGT TGA TTC-3’)<br>pINC509 (5’- ATA CTG CAG TTA TCG CCT GAC ACG ATT TCC TGC-3’)
**single-stranded ODNs**			
CpG ODN 63 (63nt)	chemical synthesis	-	5’-TCC ATG ACG TTC CTG AAT AAT TCC ATG ACG TTC CTG AAT AAT TCC ATG ACG TTC CTG AAT AAT-3’
CpG ODN 1826 (20nt)	chemical synthesis	-	5’-Fluo-TCC ATG ACG TTC CTG ACG TT-3’ (sequence from [4])

Table S3. Titration of bacteria- and yeast-produced wt HBc VLPs by ODN63.

Material/lane	***1.***	***2.***	***3.***	***4.***	***5.***	***6.***
ODN, pmol	**only empty VLPs (20 pmol)**	**200**	**400**	**600**	**800**	**1000**
ODN superiority, ratio		**10**	**20**	**30**	**40**	**50**
Contact with empty bacteria-produced HBc VLPs, ODN63 is starting to left over	**-**	**-**	**-**	**-**	+/-	+
Contact with empty yeast-produced HBc VLPs, ODN63 is starting to left over	**-**	**-**	**-**	+	+	+

Table S4. Formation of empty bacteria- and yeast-produced wt HBc VLP complexes by contact with pCEP-CXCR4-eGFP plasmid at different plasmid/VLP ratios.

Material/lane	***1.***	***2.***	***3.***	***4.***	***5.***
plasmid pCEP-CXCR4-eGFP OD_{260} / VLPs OD_{280}	**0,44**	**0,88**	**2,2**	**4,4**	**8,8**
plasmid pCEP-CXCR4-eGFP pmol / VLPs pmol	**2,8**	**5,6**	**14**	**28**	**56**
HBc VLPs superiority (over plasmid), ratio	**52**	**26**	**11**	**5**	**2,5**
Contact with empty bacteria-produced HBc VLPs, pCEP-CXCR4-eGFP is starting to left over	**-**	**-**	**-**	+	+
Contact with empty yeast-produced HBc VLPs, pCEP-CXCR4-eGFP is starting to left over	**-**	**-**	+	+	+

METHODS

Basic purification of recombinant HBc protein

To purify the wt HBc VLPs and mutant variants of HBc VLPs, 4 g of cells maintained on ice were homogenised in 12 ml of lysis buffer (50 mM Tris-HCl (pH 8.0), 5 mM EDTA, 50 µg/mL PMSF, 0.1% Triton X-100) by ultrasonification five times for 15 sec each at 22 kHz at 45 sec intervals. Next, the homogenates were supplemented with 7 M urea solution (in water) to reach a final urea concentration of 0.45 M. After 15 min, cell debris was removed by centrifugation at 12 000 rpm for 45 min, and supernatants were mixed with saturated ammonium sulphate solution to reach 1/10 of the supernatant volume. The next day, the precipitate was removed by centrifugation, and the ammonium sulphate was added to the supernatant until 60% saturation. Next, the precipitate was centrifuged and dissolved in working buffer supplemented with 0.02% Brij58 and then chromatographed on a Sepharose CL-2B (GE Healthcare Life Sciences) gel filtration column (size 60 x 2.2 cm) by eluting with working buffer supplemented with 0.02% Brij58 at a velocity of 3 ml/h (90 min/4.5 ml fraction). After optical density (OD) measurements and PAGE, VLP-containing fractions from the second peak of OD chromatogram profile were combined and precipitated with ammonium sulphate. Similarly, the precipitated VLPs were purified by re-chromatography on a Sepharose CL-4B (Sigma Aldrich) column (size 2 x 60 cm) by eluting with working buffer at a velocity of 2 ml/h (60 min/2 ml fraction), and the appropriate fractions were pooled and precipitated with ammonium sulphate. Next, the precipitate underwent solubilisation and intensive dialysis in water, followed by chromatography on a Sephacryl S-300 (GE Healthcare Life Sciences) column (1 x 50 cm) using 0.1 M Na_2CO_3, 2 mM DTT as the eluent at a velocity of 1.2 ml/h (60

min/1.2 ml fraction). VLP-containing fractions were identified using OD measurements and NAGE and pooled together. Next, an equal volume of carbonate-neutralising buffer (250 mM HEPES, 500 mM NaCl, 10 mM DTT, 300 mM $MgCl_2$, pH 4.6) was added and precipitated with ammonium sulphate. All purification steps are summarised in Supplementary Fig. S1 as PAGE images with fractions from each of used chromatography column.

Preparation of RNA samples for VLP packaging

N-Formylmethionine tRNA (or simply tRNA) was ordered to synthesise chemically; sequence of tRNA is identical as in [5].

Ribosomal RNA (rRNA) was purified from *E. coli* MRE600 or Q13 cells by grinding with glass beads in 5 mM Tris-HCl pH 7.8, 10 mM $MgCl_2$, 60 mM NH_4Cl buffer supplemented with 10 µg/ml DNAse. After the clarification of the lysate (30 min at 21000 rpm), the ribosomes were settled by ultracentrifugation for 3 h at 50000 rpm (60Ti, Spinco), and ribosomal RNA was extracted using phenol: chloroform (1:1) and ethanol precipitation. Although the rRNA was partially degraded, the quality was sufficient for the encapsidation experiments.

mRNA was transcribed *in vitro* from the linearised (with PmeI (MssI) restriction endonuclease (Thermo Scientific)) pT7CFE-Chis-DTA plasmid (see Supplementary Fig. S11a) using the TranscriptAid T7 High Yield Transcription Kit (Thermo Scientific) according manufacturer's recommendations, including DNA digestion with DNAse I and purification with phenol: chloroform extraction and ethanol precipitation. Additional purification of the poly-A fraction from total transcribed RNA pool to separate partially synthesised mRNA was performed using oligo(dT)-cellulose (Sigma-Aldrich) affinity chromatography (see Supplementary Fig. S11b).

The RNA was heated for 10 min at 70 °C, and approximately 0.04 mg of RNA per 1 mg of oligo(dT)-cellulose were mixed in binding buffer (10 mM Tris-HCl pH 7.5, 1 mM EDTA, 0.5 M NaCl) and incubated for 1 h at 4 °C. After clearing unbound RNA with binding solution, the poly(A) tail-containing mRNA was collected by washing oligo(dT)-cellulose with elution buffer (10 mM Tris-HCl pH 7.5, 1 mM EDTA) and DEPC-treated water. After the addition of RiboLock RNase Inhibitor (Thermo Scientific) to prevent degradation, the mRNA was checked for integrity in formaldehyde agarose gel electrophoresis (FAGE) (see Supplementary Fig. S11c). For a detailed FAGE protocol, view the TranscriptAid T7 High Yield Transcription Kit protocol.

Cursory evaluation of VLP nucleic acid (mononucleotide) and protein content

For precise determining of nucleic acid and protein content of viruses and VLPs by UV absorbance can be used general method elaborated by Porterfield and Zlotnick[6]. In our experiments for routine applications we used simplified calculations based on absorbance ratio at 260 nm and 280 nm for individual components – nucleotides and empty VLPs, respectively, and also for complexes. Below is an example of the calculations.

A260=a+$\boldsymbol{\beta}$b and **A280=$\boldsymbol{\alpha}$a+b**, where a is the absorption of nucleotides at 260 nm, α is the coefficient of the absorption of nucleotides at 280 nm proportionally to absorption at 260 nm (A280/A260), **$\boldsymbol{\alpha}$=0.5**, b is the absorption of protein at 280 nm, and β is the coefficient of the absorption of protein at 260 nm proportionally to absorption at 280 nm (A260/A280), **$\boldsymbol{\beta}$=0.7**.

Ratio A280/A260 for VLPs was as follows: 8.3 A260 units=1 µmol mononucleotides/ml for ssRNA, 1 A280 unit=0.71 mg for HBV core 183 protein,

A280/A260=1.42 or A260/A280=0.70 for empty VLPs, 4.8 mg VLPs correspond to 1 nmol T4 particles (0.0048 mg to 1pmol) ($183x110x240=4.83x10^6$ g/mol).

For yeast-derived core protein particles A280/A260=0.64, and for bacterial source particles A280/A260=0.62.

Calculation for yeast VLP:

1) 1=a+0.7b → a=1-0.7b
2) 2) 0.64=0.5a+b → 0.64=0.5(1-0.7b)+b → 0.64=0.5-0.35b+b → 0.14=0.65b and **b=0.215**

0.215 A280 units of HBV core protein corresponded to 0.153 mg of protein (0.215x0.71), and this amount corresponded to **31.9 pmol of T4 capsids** (0.153:0.0048)

Turn to equation 1)

a=1-0.7b → a=1-0.7x0.215=1-0.1505 and **a=0.8495**

if 8.3 A260 units are 1000 nmol mononucleotides, then 0.8495 A260 units are 102.3 nmol or 102300 pmol mononucleotides. Nucleotide content was **3208 nt/particle.**

Calculation for bacterial VLP:

1) 1=a+0.7b→ a=1-0.7b
2) 0.62=0.5a+b → 0.5(1-0.7b)+b → 0.5+0.65b

   0.12=0.65b and **b=0.1846 → 27.3 pmol of T4 capsids**

a=1-0.7b → a=1-0.7x0.1846=0.871, which corresponded to 104939 pmol of mononucleotides. Nucleotide content was **3844 nt/particle.**

Preparation of plasmid DNA, DNA fragments, and ODNs for VLP packaging

Plasmids, DNA fragments, and single-stranded ODNs used for reconstruction and contact experiments are summarised in Supplementary Table S2. Plasmids pCEP-

CXCR4-eGFP[1], pFX-GA[2] and pAS65[7] were used from previous experiments. pT7CFE1-Chis-DTA was constructed by cloning out the diphtheria toxin catalytic domain sequence (first 193 aa)[3] from commercially synthesised (GenScript) plasmid using primers pINC508 (5'-TAC ACA TAT GGG CGC TGA TGA TGT TGT TGA TTC-3') and pINC509 (5'-ATA CTG CAG TTA TCG CCT GAC ACG ATT TCC TGC-3'). After digestion with NdeI and PstI restriction endonucleases, the fragment was inserted into vector plasmid pT7CFE1-Chis (Thermo Fisher Scientific).

After transformation in *E. coli* RR1 cells, the plasmids were produced in preparative amounts and purified using the GeneJET Plasmid Maxiprep Kit (Thermo Scientific) according to the manufacturer's recommendations.

DNA fragments were acquired in different ways:

- Restriction fragments (including linearised plasmid) were cut from the appropriate plasmids using FastDigest restriction endonucleases (as shown in Supplementary Table S2) according to the manufacturer's protocol (Thermo Scientific). Preparative amounts of successfully restricted fragments were run on agarose gel electrophoresis, correct size fragments were cut from the gel using a scalpel and the DNA in the agarose was extracted using the GeneJET Gel Extraction Kit (Thermo Scientific) according to the manufacturer's protocol.
- PCR fragments were synthesised using PCR Master Mix (2×) (Thermo Scientific) with the addition of appropriate template DNA (plasmid final concentration 0.5 μg/ml) and primers (final concentration 0.2 mM). All primers (including single-stranded ODNs in Supplementary Table S2) were ordered from Metabion. Preparative amounts of PCR fragments were also purified through agarose gel electrophoresis as restriction fragments.

- Single-stranded ONDs were ordered from Metabion and used after reconstitution in water.

Next Generation Sequencing (NGS)

Sequencing was conducted using the Life Technologies Ion Torrent™ PGM platform, which is based on semiconductor sequencing technology[8]. Sequencing was performed for mRNA and materials extracted from bacteria-produced wt HBc VLPs and from contact packaging of HBc-K80 VLPs with mRNA. Samples were fragmented before library construction.

Library construction. The Invitrogen Qubit 2.0 Fluorometer (Invitrogen) was used to normalise the RNA concentration to 20 ng/µl. A modified barcoded RNA library was prepared using the Ion Total RNA-Seq Kit (Publication part Nr. 4467098 Rev C, Revision Date October 2011). RNA fragmentation was conducted using the Covaris S220 focused-ultrasonicator (Covaris) with shearing parameters for 200 bp long DNA fragments: peak incident power – 175; duty factor - 10%; cycles per burst – 200; treatment time - 240 s; temperature – 7 °C; water level S220 – 12; sample volume - 50 µl. The size and yield of the fragmented RNA was assessed using the Agilent RNA 6000 nano Kit. In total, 3 µl (100 ng/µl) of fragmented RNA, 1 µl of Ion Xpress RNA Seq Barcode, and 1 µl of Ion Xpress RNA 3' Adaptor were mixed with 3 µl of hybridisation solution and incubated at 65 °C for 10 min and then at 30 °C for 5 min. After hybridisation, 10 µl of 2x Ligation Buffer and 2 µl of Ligation Enzyme Mix was added to the sample following incubation at 30 °C for 30 min. Reverse transcription, further purification and library amplification steps were performed as in the standard protocol. The final library quantification and quality check was performed on the

Agilent 2100 Bioanalyser using High Sensitivity DNA chips (Agilent Technologies). The libraries were diluted to approximately 26 pM.

Sequencing. The Ion OneTouch™ 200 Template Kit v2 DL (Release: 12 September 2012, Publication Nr. MAN0006957) was used for template preparation and emulsion PCR. The Ion PGM™ 200 Sequencing Kit (Publication Nr. 4474246, Rev H) and standard sequencing protocol for the Ion 314™ chip type were used for the sequencing procedure.

Data analysis. Quality control was performed during the base calling step. The threshold for the average quality values within the window of size 30 was set to 15. The adapter trimming cutoff was set to 16. The reads were mapped against plasmids pHBc183 and DTA and the *E. coli* CFT073 genome (GenBank accession number AE014075.1) with Torrent Mapping Alignment Program (tmap 3.4.1) using the k-mer lookup algorithm (mapping procedure #3). The resulting bam files were sorted and indexed with samtools version 1.0-11-geeb4b22 (using htslib 1.0-2-g1903fd4). The coverage of reads mapped against the reference genomes was calculated with bedtools v2.17.0 genomecov tool using the -bg option. The number of total mapped nucleotides and number of mapped nucleotides for each reference genome were calculated using perl script by multiplying coverage (4th column in bed file) with the span of the coverage (start coordinate subtracted from end coordinate). Additionally, the amount of unmapped nucleotides was estimated by counting all nucleotides in the unmapped reads.

REFERENCES

1 Strods, A., Petrovska, R., Jackeviča, L. & Renhofa, R. Cloning and expression of chemokine receptor CXCR4 in eukaryotic cells CHO, HEK293 and BHK21. *Proceedings of the Latvian Academy of Sciences. Section B. Natural, Exact, and Applied Sciences.* **64**, 98–105, doi:10.2478/v10046-010-0033-6 (2011).

2 Freivalds, J., Rūmnieks, J., Ose, V., Renhofa, R. & Kazāks, A. High-level expression and purification of bacteriophage GA virus-like particles from yeast Saccharomyces cerevisiae and Pichia pastoris. *Acta Univ. Latv.* **745**, 75–85 (2008).

3 Greenfield, L. *et al.* Nucleotide sequence of the structural gene for diphtheria toxin carried by corynebacteriophage beta. *Proc Natl Acad Sci U S A* **80**, 6853-6857 (1983).

4 Storni, T. *et al.* Nonmethylated CG motifs packaged into virus-like particles induce protective cytotoxic T cell responses in the absence of systemic side effects. *J Immunol* **172**, 1777-1785 (2004).

5 Gauss, D. H., Gruter, F. & Sprinzl, M. Compilation of tRNA sequences. *Nucleic Acids Res* **6**, r1-r19 (1979).

6 Porterfield, J. Z. & Zlotnick, A. A simple and general method for determining the protein and nucleic acid content of viruses by UV absorbance. *Virology* **407**, 281-288, doi:10.1016/j.virol.2010.08.015 (2010).

7 Strods, A., Argule, D., Cielens, I., Jackeviča, L. & Renhofa, R. Expression of GA Coat Protein-Derived Mosaic Virus-Like Particles in Saccharomyces cerevisiae and Packaging in vivo of mRNAs into Particles. *Proceedings of the Latvian Academy of Sciences. Section B. Natural, Exact, and Applied Sciences* **66**, 234–241, doi:10.2478/v10046-012-0015-y (2013).

8 Rothberg, J. M. *et al.* An integrated semiconductor device enabling non-optical genome sequencing. *Nature* **475**, 348-352, doi:10.1038/nature10242 (2011).
